# Changes in the cortical GABAergic inhibitory system in a Spinal Muscular Atrophy mouse model

**DOI:** 10.1038/s41419-026-08520-8

**Published:** 2026-02-28

**Authors:** Giovanna Menduti, Francesco Ferrini, Anna Caretto, Amber Hassan, Raffaella di Vito, Giada Beltrando, Davide Marnetto, Alessandro Usiello, Ferdinando Di Cunto, Marina Boido, Alessandro Vercelli

**Affiliations:** 1 Neuroscience Institute Cavalieri Ottolenghi, Orbassano, Turin, Italy; 2 Department of Neuroscience “Rita Levi Montalcini”, University of Turin, Turin, Italy; 3 Department of Veterinary Sciences, University of Turin, Grugliasco, Turin, Italy; 4 Department of Psychiatry and Neuroscience, Université Laval, Québec, QC Canada; 5 European School of Molecular medicine, University of Milan, Milan, Italy; 6 Laboratory of Translational Neuroscience, Ceinge Biotecnologie Avanzate, Naples, Italy; 7 Department of Environmental, Biological and Pharmaceutical Science and Technologies, Università degli Studi della Campania “Luigi Vanvitelli”, Caserta, Italy

**Keywords:** Cell death in the nervous system, Neurodegeneration, Molecular neuroscience, Cellular neuroscience, RNA splicing

## Abstract

The cortical motor network excitatory-inhibitory (E/I) imbalance contributes to several neurodegenerative movement disorders. Spinal Muscular Atrophy (SMA) is a neuromuscular disease due to the lack of Survival Motor Neuron (SMN) protein, characterized by lower motor neuron (MN) degeneration and muscle atrophy. However, evidence shows that SMA patients display motor cortex abnormalities correlating with disease severity, suggesting altered maturation and maladaptive plasticity potentially contributing to upper MN vulnerability. This raises questions about cortical involvement and highlights the need for preclinical studies to clarify underlying mechanisms, given the limited accessibility of early-stage, untreated brain tissue from SMA patients. In agreement, our previous work in SMA mice revealed upper MN vulnerability, indicating SMA pathogenesis is far more complex than classically conceived. Here, by employing a combination of imaging, molecular techniques, and electrophysiological characterization of cortical inhibitory neurotransmission, we dissected GABAergic signalling, metabolism, and interneuron function in the sensorimotor cortex and primary neuron-astrocyte co-cultures of a severe SMA mouse model. Additionally, we conducted bioinformatic analyses and biochemical assays to assess age-dependent modulation of neurotransmitter pathways and quantify key metabolites across different stages of the disease, with the overall aim of evaluating correlations between GABA levels, its precursor glutamine, the expression of synthetic enzymes (GAD65/67), and the density of Parvalbumin-positive interneurons with SMN deficiency. We unveiled a significant association between SMN deficiency and impaired density, morphology and signalling of GABAergic Parvalbumin positive interneurons in the sensorimotor cortex of late-stage SMA mice, suggesting E/I imbalance and possibly contributing to shape upper MN vulnerability. We also highlighted the pivotal role of SMN, as involved in pre-mRNA splicing, in its impact on neuronal-astrocyte interactions regulating GABA metabolism, release and reuptake. These findings underscore a role for altered motor cortical GABAergic neurotransmission in SMA progression and offer a new key perspective to achieving novel, comprehensive therapeutic approaches.

## Introduction

Several neurodevelopmental, neuropsychiatric and neurological diseases are characterized by cortical excitatory-inhibitory (E/I) imbalance [1]. In neurodegenerative movement disorders [as Parkinson’s disease (PD), Huntington’s disease (HD) or Amyotrophic lateral sclerosis (ALS)], such alterations specifically affect cortical motor areas [1–6]. The E/I imbalance can determine large-scale network dysfunctions and seems more and more implicated in the deterioration of symptomatology of many diseases, including motor neuron diseases (MNDs) [1, 5]. Spinal muscular atrophy (SMA) is a paediatric MND and the leading genetic cause of infant mortality. Due to mutation of the Survival Motor Neuron 1 (*SMN1*) gene, SMA is characterized by the SMN protein deficit, resulting in motor neuron (MN) impairment, skeletal muscle atrophy and premature death [7, 8]. Although SMN deficiency primarily affects lower MNs, its ubiquitous depletion dramatically affects cells and organs [9]. Supraspinal neuropathological findings have been reported in patients with severe SMA, overall suggesting that the lack of SMN has widespread consequences and manifestations in the central nervous system (CNS) that are not limited to lower motor neurons (LMNs) [10–12].

Recent neuroimaging and neurophysiological studies indicate that the motor cortex (CRTX) is both structurally and functionally affected in SMA patients, with variations in cortical changes correlating with disease severity and phenotype, suggesting the coexistence of compensatory and maladaptive plasticity alongside neurodegeneration. In detail, early transcranial magnetic stimulation studies in SMA types 2 and 3, employing threshold-tracking techniques and normalization of muscle responses to peripheral stimulation, quantified and showed preserved motor cortical output alongside disproportionately increased muscle responses to cortical stimulation, suggesting compensatory neuroplasticity [13]. However, more recent electroencephalography-electromyography coherence studies reveal altered corticospinal interactions and recruitment of broader cortical networks, suggesting functional reorganization of the motor CRTX [14]. Structurally, magnetic resonance imaging (MRI) studies reveal phenotype-dependent cortical changes: milder forms (SMA 3 and 4) show increased grey matter density in motor cortical areas in comparison to healthy controls, possibly reflecting compensatory or maladaptive plasticity [15], with greater grey matter volume correlating with longer disease duration and slower progression. In contrast, studies in SMA type 2 patients exhibit reduced cortical thickness in the precentral gyrus—including the primary motor CRTX (M1)—that correlates with motor decline, indicating neurodegeneration [16]. Notably, histopathological data from SMA type 2 autopsies support and may help explain, at least in part, the MRI findings by showing cortical microstructural changes such as loss of large pyramidal neurons (Betz cells) in the M1 and reduced myelinated fibers in corticospinal pathways [17]. Furthermore, severe prenatal-onset SMA type I cases reveal pronounced cortical involvement in the disease process, with degenerative changes in layer V of the M1 characterized by chromatolytic ballooned neurons and neuronophagia [12].

Taken together, these findings suggest that SMA involves complex motor cortical changes rooted in altered maturation and remodelling of corticospinal circuits. Such changes may partly reflect an E/I imbalance involving motor cortical neurons and interneurons (INs) that shape circuit remodelling, potentially contributing to upper MN vulnerability, though these mechanisms warrant further investigation.

In parallel, mechanistic insights from animal models support these observations. Morphological and developmental alterations have been observed in the M1 of SMA murine models, including a selective degeneration of layer 5 pyramidal neurons and differences in intrinsic synaptic and neuronal properties, from pre-symptomatic to late symptomatic disease stages [10, 18, 19].

Nowadays, the available treatments effectively increase the SMN level and delay disease progression, but do not yet represent a cure for SMA due to many intrinsic limits [20]. Among them, limited effects when lately administered and still unknown long-term efficacy in ensuring ubiquitous and permanent restoration of endogenous SMN expression underline the need for complementary therapies [21, 22]. In this context, antiepileptic and antipsychotic drugs (AEDs and APDs) proved to prevent deterioration of motor functions and attenuate SMA disease progression [20, 23]. These drugs act primarily on impaired brain E/I, thus limiting excitotoxicity and the subsequent neuronal death. Their mechanism of action relies either on: i) modulating voltage-gated ion channels, affecting excitatory neurotransmission [e.g. valproic acid, lamotrigine and riluzole [24–26]]; or on ii) enhancing and preventing, directly or indirectly, dysregulations in γ-aminobutyric acid (GABA)-mediated inhibitory neurotransmission [e.g. gabapentin, gaboxadol, levetiracetam [20, 27–30]. Intriguingly, the GABAergic dysregulations, a neurological hallmark of epilepsy and neuropsychiatric disorders [30, 31], can also impact the activity of the motor cortical network in MND, such as ALS [1, 5]. In SMA, although SMN deficiency is associated with spinal MN hyperexcitability [32–34], and recent work has further demonstrated that spinal circuit alterations involve a maladaptive shift in the E/I balance, where excessive inhibitory drive exacerbates motor impairment [35]. However, it remains unclear whether, and to what extent, this phenomenon might be associated with E/I dysfunction within cortical motor networks, thus supporting the beneficial treatment effects of AEDs/APDs. Thus far, GABA signalling and its effects on molecular pathways and altered functions of IN populations at the cortical level, such as their correlation with SMN deficiency in the disease onset and progression, have been largely neglected. GABAergic dysfunctions, whether in IN density or in their regulatory role on cortical output, may contribute to, and partially underlie, the connectivity and structural alterations observed in the motor cortex of SMA patients.

Here we report our studies on GABAergic signalling and metabolism, as well as IN distributions and function in sensorimotor [SM, i.e., M1 and somatosensory (S1)] cortex (CRTX) of SMAΔ7 mice, a severe SMA murine model [36]. To this aim, biochemical detection assays, immunofluorescence staining and molecular analysis of GABA, its precursors, synthetic enzymes and Parvalbumin (PV)-positive INs were performed in SMA cortical samples at the pre-symptomatic [postnatal day (P)1], early (P5), late disease stage (P12) as well as in primary neurons and astrocytes co-cultures, in comparison with WT controls. Cortical GABAergic inhibitory neurotransmission was also electrophysiologically characterized and correlated with SMN deficiency. To the best of our knowledge, this study is the first to pinpoint and shed new light on SMA-related GABAergic dysregulation at the cortical level, and on the key role of SMN deficiency in influencing E/I neurotransmission.

## Materials and Methods

### Experimental design

We investigated GABAergic signalling in the sensorimotor cortex of SMAΔ7 mice (from now on SMA), a severe SMA murine model [36], at the late stage of the disease. We quantified GABAergic synthesis markers [glutamic acid decarboxylase (GAD) enzymes] and signalling markers (PV+ INs) expression in the SM CRTX using molecular (immunoblotting) analyses and analysed their distribution and morphology in different cortical areas with various imaging techniques. To rule out the possibility that the expression and distribution of GABAergic markers could be compromised or dysregulated due to neurodevelopmental issues or during the earlier stages of the pathology, we conducted bioinformatic analyses and biochemical assays (HPLC analysis) to assess neurotransmitter pathway modulation and levels across different ages. Synaptic neurotransmission was assessed through morphological and electrophysiological (Patch Clamp) analyses. Additionally, we studied GABAergic signalling in co-cultures of primary mouse neurons and astrocytes in SMA and WT samples (also by means of RNAi to reduce SMN expression in WT controls) and performed immunohistochemistry to assess neurotransmitter dysregulation at the single-cell level. Finally, we analysed the correlation between dysregulated GABAergic pathways and SMN deficiency in disease conditions, compared to WT controls.

All the ex vivo and in vitro experiments have been carried out by blinded researchers in a randomized manner. Animals were randomly assigned to experimental groups to minimize allocation bias. Randomization was not performed using an automated or algorithmic approach. An a-priori power analysis was conducted using G*Power software, informed by the variability observed in our prior studies with the same mouse strain and experimental techniques [8, 37–39]. All procedures adhered to ARRIVE guidelines and were repeated at least three times. Replicability was confirmed across individual mice and cell cultures, with data points included if they met technical validity standards (e.g., perfusion, tissue preparation, staining, recordings, imaging). A total of 82 mice (41 SMA, 41 WT) were analyzed using immunofluorescence, molecular, and electrophysiological techniques. The experimental procedures with live animals were performed in strict accordance with institutional guidelines in compliance with national (D.L. N.26, 04/03/2014) and international law and policies (new directive 2010/63/EU). The study was approved by the Italian Ministry of Health (protocol #160/2020-PR). Additionally, the ad hoc Ethical Committee of the University of Turin approved this study.

**Supplementary Materials and Methods** for this manuscript are available as part of the Supplementary material. This includes sections related to animals, tissue collection and processing, HPLC analysis in mice CRTX samples, immunofluorescence assays on brain sections and primary cortical cells, imaging and analysis, immunoblotting analysis, bioinformatic analysis, RNAi SMN expression and Nusinersen treatment in primary cortical neurons and declaration of LLM tools technologies in the writing process.

Additionally, the Supplementary Information includes Table S1, listing all primary and secondary antibodies used in immunofluorescence and immunoblotting experiments, as well as the full bibliography for the Supplementary Materials and Methods.

### Statistical analysis

All the experiments' statistical details are reported in the figure legends, including the statistical tests used, the sample size (*n*) and *p*-value. All statistical analyses were performed using GraphPad Prism 8.0 software (GraphPad software, San Diego, CA, USA). In immunofluorescence and immunoblotting analyses, parametric data were analysed by unpaired two-tailed Student’s *t* test, by ordinary OneWay ANOVA or Two-way ANOVA, followed by Sidak’s multiple comparison post hoc test. For HPLC analysis, the normal data distribution was checked using the Shapiro-Wilk and Kolmogorov-Smirnov s tests. Then, an unpaired Student’s *t*-test was used to compare the amino acid level in SMA mice with WT littermates. For primary cortical cell and synaptic puncta analysis, data were analysed by the unpaired Mann-Whitney test. Values of *p* < 0.05 were considered statistically significant (*p < 0.05; **p < 0.01; ***p < 0.005; ****p < 0.001). In electrophysiology experiments, interevent interval distribution per each neuron before and after TTX was compared by the Kolmogorov-Smirnov test (considered as significant if *p* < 0.01). Pooled data of IPSC frequency and amplitude were compared either by paired Student’s *t* test or by Two-way ANOVA followed by Fisher’s post-hoc test for multiple comparisons (*p < 0.05; **p < 0.01; ***p < 0.005; ****p < 0.001). For the correlation analysis of neuronal fluorescent signal data in primary cortical neurons (Fig. S7), Pearson's r correlation analysis was performed. (****p < 0.001).

## Results

### Neuronal GABAergic signalling is impaired in SMA motor cortex and primary cortical cultures

**Fig. 1:**
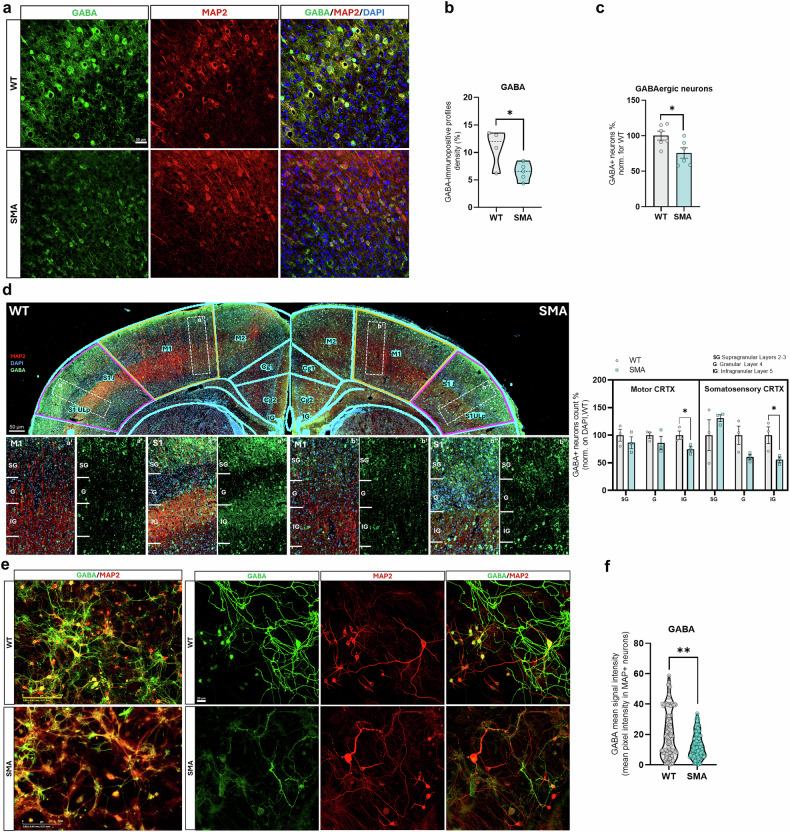
Neuronal GABAergic signal is impaired in both SMAΔ7 mice, SM CRTX and primary cortical cultures. **a** Representative immunofluorescence (IF) images of neurons co-labelled for GABA (green) and MAP2 (red) in WT and SMA mice SM CRTX at P12. Nuclei are DAPI-stained (blue). Scale bar: 30 µm. **b** Analysis of GABA-immunopositive profiles density in WT and SMA SM CRTX (Student’s t-test, *p < 0.05; each dot represents the mean value from an individual animal, violin plots show the distribution of these values across animals within each group, including median and variability; WT mice: *n* = 4; SMA mice: *n* = 5). **c** Percentage of SMA GABA + SM CRTX neurons (Data are presented as mean ± SEM, Student’s t-test, *p < 0.05; WT and SMA mice: *n* = 6). **d** Representative immunofluorescence (IF) images of coronal brain sections co-labelled for GABA (green) and MAP2 (red) in WT and SMA mice SM CRTX. Nuclei are DAPI-stained (blue) from WT and SMA mice. Brain atlas outlines are superimposed (cyan) to highlight the primary motor (M1, orange) and somatosensory (S1, magenta) cortical areas. Scale bar: 50 µm. Higher-magnification views of the boxed regions show M1 (a′, b′) and S1 (a″, b″) cortical areas in WT and SMA mice, respectively. GABA immunoreactivity (green) is shown within cortical layers [supragranular (SG), granular (G), and infragranular (IG)]. Quantification of GABA⁺ cell density (%) in M1 and S1 regions is reported in the right panel, comparing SMA mice with WT controls (data are expressed as mean ± SEM and normalized to WT mean, referred to as control; Student’s t-test, *p < 0.05; WT and SMA mice: *n* = 3). **e** Representative widefield (left) and confocal (right) immunofluorescence images of WT and SMA primary cortical neurons at *DIV 15*. Scale bar: 200 and 30 µm. **f** Quantification of GABA mean intensity in MAP2+ neurons among WT and SMA cortical cells; analyses included at least 100 cells from six independent cultures for each group. Each dot on the graph represents an individual cell; violin plots show the distribution of these values within each group, including median and variability (Mann-Whitney test, **p < 0.01; WT and SMA mice: *n* = 6 primary cell co-cultures).

To assess the involvement and possible dysfunctions of GABA pathways in the SMA cortical motor network, first we evaluated whether the GABA signal intensity and the number of GABA+/MAP2+ neurons were changed in SM CRTX samples from P12 SMA mice compared to WT controls (Fig. 1a). We observed a significant reduction in GABAergic immunopositive profile density (WT: 10.93% ± 1.69, SMA: 6.45% ±0.70; *p* < 0.05) (Fig. 1b) and a lower number of GABA+ neurons in SMA SM CRTX (−38% ± 6.47, *p* < 0.05) compared to WT (Fig. 1c), suggesting a decrease in neurotransmitter levels or distribution in these cortical regions in late disease stage. By counting GABA⁺ neurons across different layers of the SM CRTX (Fig. 1d), we found an overall reduction in the number of inhibitory neurons in SMA, with a significant loss in cortical layer 5 (M1: –25% ± 9.09, p < 0.05; S1: –44% ± 15.83), consistent with a selective vulnerability of output-layer circuits rather than a pan-cortical degeneration (Fig. 1d).

Reduced GABA immunopositive signal was also confirmed at the single cell level in primary cultures of cortical neurons from SMA and WT mice (WT neurons: 20.21 ± 1.52; SMA neurons: 12.54 ± 0.78, *p* < 0.01; Fig. 1e-f). Additionally, the GABA signal was assessed using two complementary measures: the percentage of GABA⁺ area relative to the total field and the percentage within MAP2⁺ neurons. Both analyses indicated an approximately 8% reduction in SMA cultures compared to WT, whereas the proportion of GABA⁺ neurons within the MAP2⁺ population (~25%) remained similar across groups (Figure S1a–c).

Taken together, these data support the interpretation that the reduction in GABA signal in SMA is best explained by functional and circuit-level alterations at late-stage disease. To rule out a global depletion of cortical GABA, we performed HPLC on whole-cortex homogenates as a conservative control for overall neurotransmitter levels. In parallel, we performed biochemical quantification assays of GABA levels (together with other related neuroactive amino acids, such as L-Glu and L-Gln) in the whole CRTX of SMA mice compared to WT controls, at P1, P5 and P12.

**Fig. 2:**
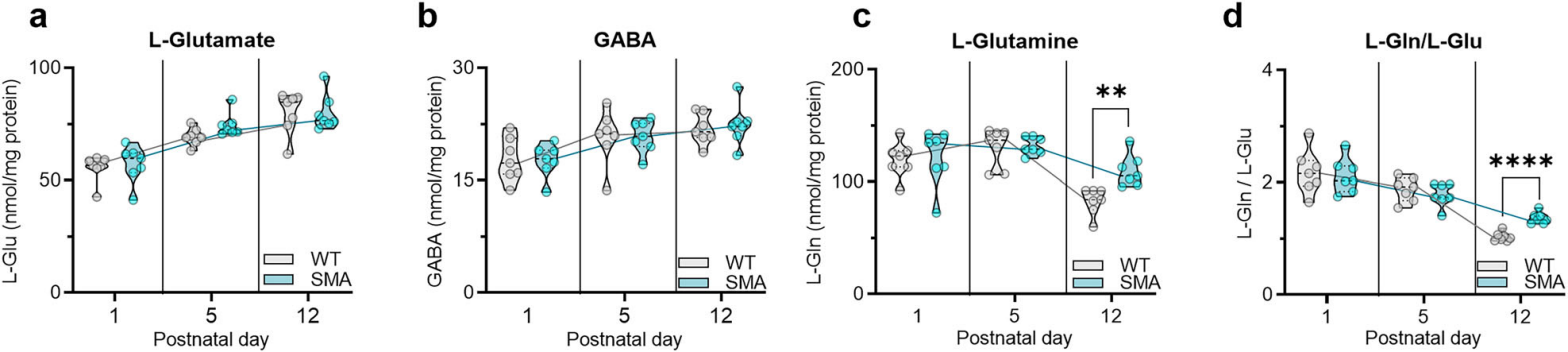
Cortical amino acid profile is altered in SMA mice at the late disease stage. Analysis of free L-Glutamate (Glu) (**a**), GABA (**b**), L-Glutamine (Gln) (**c**) levels and L-Gln/L-Glu ratio (**d**) in the CRTX of SMA mice in comparison to WT controls by HPLC analysis in different mice ages (P1, P5, P12). In each sample, all free amino acids were detected in a single run (Student’s *t*-test, **p < 0.01, ****p < 0.001; WT and SMA mice: *n* = 7).

Results showed that Glu and GABA levels are not apparently affected in the whole CRTX of SMA mice at the different time points (Fig. 2a, b), while Gln and L-Gln/L-Glu ratio are increased in the SMA mice CRTX at the late symptomatic stage (L-Gln: 26.97 ± 6.96, *p* = 0.0022; L-Gln/L-Glu: 0.33 ± 0. 047, *p* < 0.0001) (Fig. 2c, d). These findings highlighted two key perspectives to further analyse GABAergic system in SMA disease: first, they excluded a chemical dysregulation in the main inhibitory and excitatory neurotransmitter levels in the whole SMA CRTX, suggesting a selective vulnerability of the SM cortical areas to GABA dysregulation; second, they highlighted, intriguingly only in SMA late disease stage, the cortical accumulation of Gln, which serves as a neuronal precursor for the de novo synthesis of Glu and, subsequently, GABA [40].

**Fig. 3:**
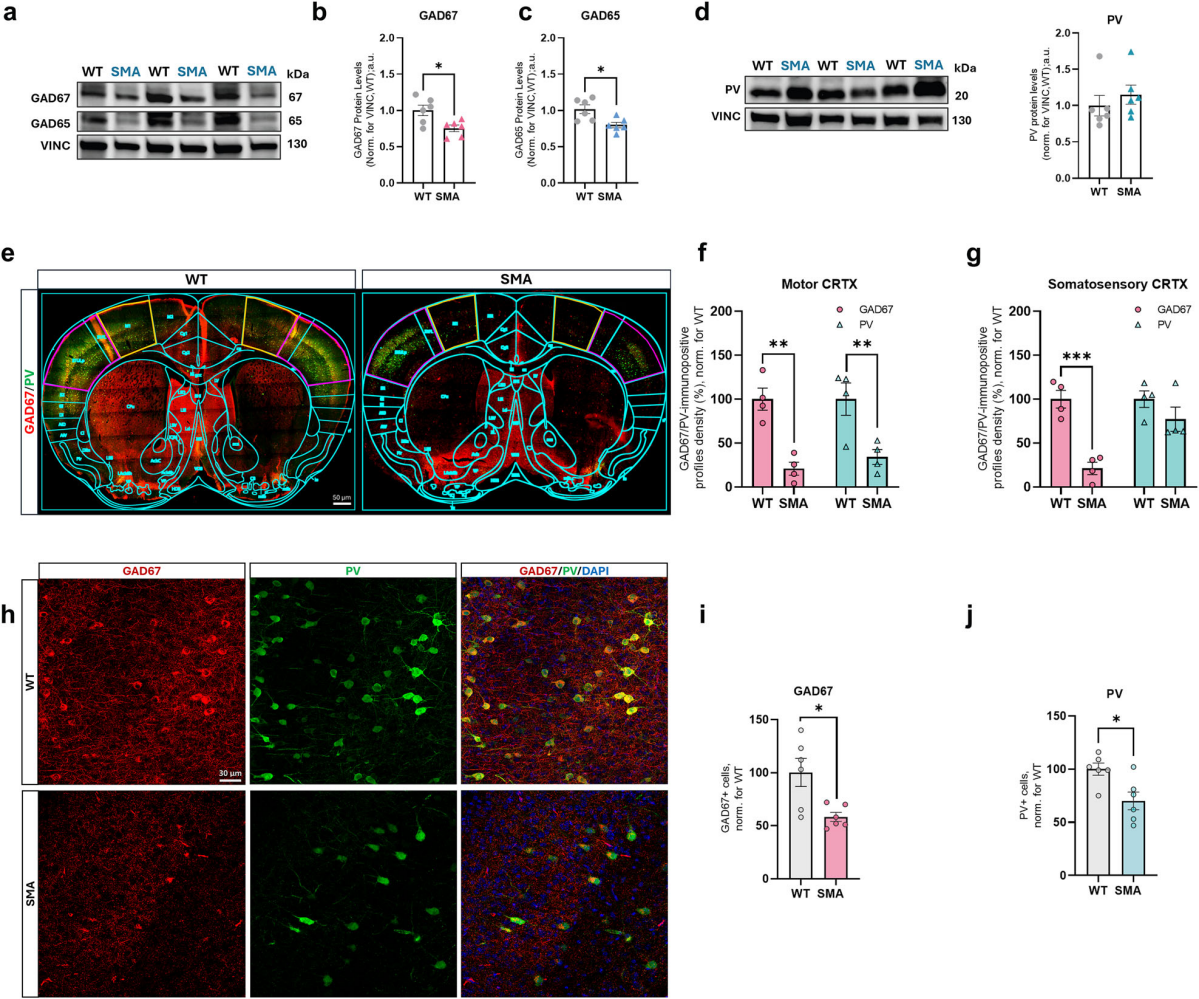
GABAergic signal and metabolism are impaired in SMAΔ7 mice SM CRTX. **a** Immunoblotting analysis of GABAergic molecular marker expressions in P12 SMA and WT SM CRTX. Representative densitometries and protein levels quantifications of GAD enzymes (**a**–**c**) and PV (**d**) in SMAΔ7 SM CRTX homogenates compared to WT controls [Data are expressed as mean ± SEM and normalized to VINC (referred as loading control) and to WT mean. Student’s t-test, *p < 0.05; WT and SMA mice: *n* = 6]. **e** Representative IF images of coronal brain sections from WT and SMA mice. Related brain atlas table is superimposed (cyan), highlighting M1 (orange) and S1 (magenta) areas. Scale bar: 50 um. Herein, analysis of GAD67+ and PV + IN cell signal (%) were performed in motor (**f**) and somatosensory (**g**) areas of SMA mice in comparison with WT ones (data are expressed as mean ± SEM and normalized to WT mean, referred as control; Student’s t-test, **p < 0.01, ***p < 0.001; WT and SMA mice: *n* = 4). **h** Representative confocal images of GAD67+ (red) and PV+ cells (green) in WT and SMA mice SM CRTX. Nuclei are DAPI-stained (blue). Scale bar: 30 um. Quantification of GAD67+ (**i**) and PV + (**j**) neuronal cell density in the SM CRTX (data are expressed as mean ± SEM and normalized to WT mean, referred as control; Student’s *t*-test, *p < 0.05, ***p < 0.001; WT and SMA mice: *n* = 6).

To further extend these analyses, we dissected GABA metabolism and IN features by focusing on the expression of GAD65 and GAD67 (the GABA synthesis enzymes) and of the calcium-binding protein PV in INs and the immunoreactivity and the density of GAD67+ and PV+ cells in SM CRTX of P12 SMA mice compared to WT controls [31, 41–44]. By WB, a significant reduction in protein levels of the GAD67 and GAD65 enzymes (−25% ± 8.51, *p* < 0.05 and −22% ± 7.04, *p* < 0.05, respectively) was observed in the SMA SM CRTX (Fig. 3a–c) compared to controls. To further investigate the temporal onset of these alterations, we analysed GABAergic pathway markers in the SM CRTX at P5, corresponding to the early symptomatic stage. At this stage, cortical GABAergic neurons are still undergoing the developmental “GABA switch” [10, 45–49], during which GABA transmission shifts from excitatory to inhibitory. No changes in GAD65 or GAD67 protein levels were observed at P5, indicating that the deficits detected at P12 emerge after the GABA switch and are not due to early developmental defects, in line with the previous biochemical analyses (Fig. S2a).

The analysis of PV protein levels at P12 apparently did not show differences between SMA and WT mice (Fig. 3d). However, by analysing the cell profile density by IF distinguishing between M1 and S1 (Fig. 3e), we showed a concomitant significant reduction of GAD67 and PV immunopositive profile in SMA M1 (GAD67: −79.13% ± 17.66, *p* < 0.01; PV: −65.81% ± 17.66, *p* < 0.01) (Fig. 3f). A similar significant reduction of GAD67 immunopositive profile density was also found in S1 (GAD67: −78.80% ± 14.65, *p* < 0.005) (Fig. 3g), while, despite a decreasing trend, no significant reduction in PV+ profiles was detected in the same area. Additional confocal analyses, enabling manual counting of neurons positive for the selected GABAergic markers, confirmed a significant reduction of GAD67+ cells (−42% ± 4.26, *p* < 0.05) and of PV+ INs (−30% ± 8.29, *p* < 0.05) (Fig. 3h–j). Also, GAD65 immunopositive profile density was analysed in the same cortical regions, highlighting a decrease in both M1 (−71% ± 20.96, *p* < 0.05) and in S1 regions (−48% ± 32.73, *p* = 0.21) (Fig. S3). The GAD65 enzyme regulates the phasic, activity-dependent synthesis of GABA and its release within nerve terminals and synapses, aiding in the packaging of the neurotransmitter into vesicles by forming a complex with the vesicular GABA and glycine transporter (VGAT) [50]. Given the significant reduction of the GAD65 enzyme expression and distribution in SMA SM CRTX, we aimed to evaluate whether VGAT protein levels should also be dysregulated. Intriguingly, the immunoblotting analysis highlighted a significant increase in VGAT protein levels (+18% ± 7.19, *p* < 0.05) in SMA mice compared to controls (Fig. S4), suggesting a differential regulation of the GABA/glycine vesicular transporter in the SMN deficiency condition as compared to GAD enzymes. VGAT expression analysis at the early symptomatic stage (P5) also revealed a modest but statistically significant increase in SMA mice (+10% ± 4.03, p < 0.05), suggesting that this upregulation emerges early in disease progression and may contribute to functional compensation **(**Fig. S2b).

Overall, these findings suggest a reduction in GABAergic signal intensity in SMA cortical neurons, which may reflect alterations in neuronal GABA metabolic pathways at the cortical level.

### GABAergic synapses and activity-dependent inhibitory neurotransmission are impaired in the SMA SM cortex

**Fig. 4:**
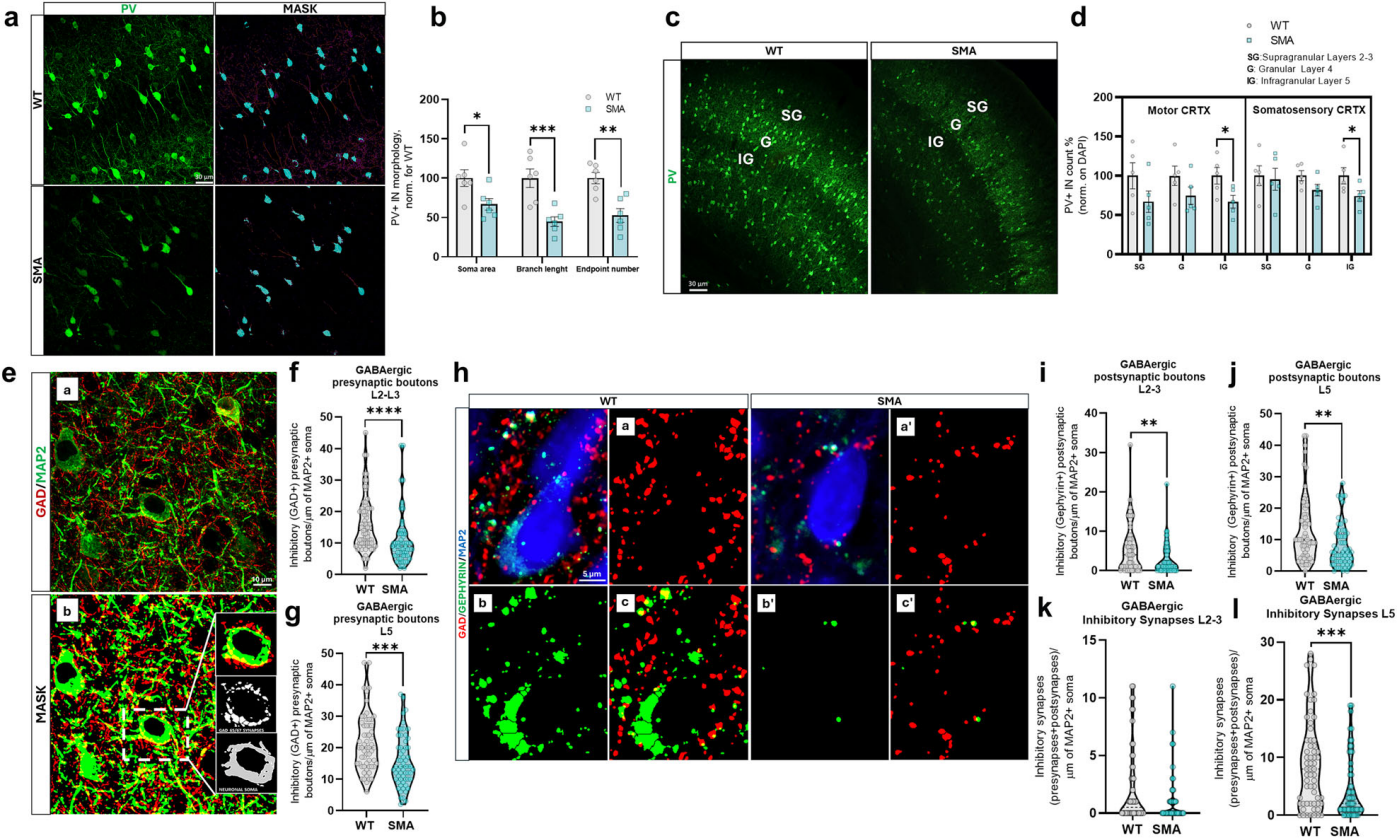
GABAergic PV + IN and synapses are impaired in SMAΔ7 SM cortical layers. **a** Confocal analysis of PV+ INs in mice SM CRTX (Scale bar: 30 µm) with semi-automated segmentation of PV+ cells (green). The binary mask of soma (cyan) and neurite skeleton (red/magenta) (**c**) was processed for IN shape quantitative analysis (**b**). Morphological PV + IN measurements are showed for SMA vs. WT (data are expressed as mean % ± SEM and normalized to WT mean; Student’s *t*-test, *p < 0.05, ***p < 0.001; WT/ SMA mice: *n* = 6). **c** Representative IF images of PV+ INs in mice SM CRTX layers. Scale bar: 30 um. **d** PV + IN count performed in mice M1/S1 layers (data are expressed as mean ± SEM; Two-way ANOVA, followed by Sidak’s multiple comparison post hoc test, *p < 0.05; WT /SMA mice: *n* = 5). **e** Confocal frames in SMA a*n*d WT motor CRTX layers (Scale bar: 30um) were processed to create a binary mask for GABAergic+ synapses (GAD65 + 67, red) and neuronal soma (MAP2, green). Inhibitory pre-synapse boutons were quantified based on co-localization around the neuronal soma perimeter. Violin plots show the cumulative distribution of GABAergic synapses contacting-neurons in L2/3 (**f**) and L5 (**g**) (Mann-Whitney test, ***p < 0.005, ****p < 0.001; each dot on the graph represents the number of GABAergic synapses from an individual neuron; at least 60 neurons were analyzed from three different animals per group). **h** Single optical plane analysis in SMA and WT motor CRTX layers (Scale bar: 5 µm). Images were processed to create binary masks for (a,a’) GABAergic pre-synapses (GAD + , red), (b,b’) GABAergic post-synapses (gephyrin + , green), and neuronal soma (MAP2 + ). Inhibitory synapse complexes (GAD+ and gephyrin + ) were quantified based on co-localization around the neuronal soma perimeter (c,c’). Violin plots show the cumulative distribution of Gephyrin + synapses contacting-neurons in L2/3 (**i**) and L5 (**j**) and inhibitory synapse complexes in L2/3 (**k**) and L5 (**l**) (Mann-Whitney test, **p < 0.01; ***p < 0.005; ****p < 0.001; each dot on the graph represents the number of GABAergic synapses from an individual neuron; violin plots show the distribution of these values across animals within each group, including median and variability; at least 60 neurons were analyzed from three different animals per group).

As reduced levels of GAD67 can correlate with morphological alterations of PV+ INs [41–44], the latter were assessed in SMA cortical samples (Fig. 4a). Confocal analysis highlighted significantly smaller somas (-33% ± 7.38, *p* < 0.01), reduced length (-55% ± 6.15, *p* < 0.005) and lower number of dendritic branches (−47% ± 8.71, *p* < 0.01) of the PV+ INs in the SM CRTX of SMA mice compared to controls (Fig. 4b). While confirming the previous observations, these results also suggest possible defective inhibitory functions of PV+ INs: their morphological alterations in SMA may negatively influence their ability to effectively connect to excitatory neurons and carry out their inhibitory filtering and synchronization of activities [51, 52]. Therefore, to better circumstantiate these results, additional experiments were carried out to evaluate whether the decreased number of PV+ INs in SMA SM CRTX could affect a specific SM cortical layer.

As known, cortical INs modulate the pyramidal neuron activity, depending on their position, distribution in the different layers, the spatial distribution of their axonal arborizations and on their pattern of connectivity [53]. Therefore, we specifically investigated the PV + IN distribution in M1 and S1 cortical areas of SMA mice, by quantifying their density in different cortical layers in comparison with WT (Fig. 4c). Results highlighted an overall reduction of PV+ INs in layers 2/3, 4 and 5 of both M1 and S1 areas of SMA mice compared to controls. Intriguingly, such reduction was statistically significant in layer 5 of both the M1 (−33.07% ± 13.32, *p* < 0.05) and S1 CRTX (−25.70% ± 12.32, *p* < 0.05) (Fig. 4d). These data support the hypothesis of a SMA-associated PV + IN dysregulation, also resulting in alterations of their distribution between the cortical layers (significant at layer 5), thus suggesting a possible loss of the GABAergic contribution to cortical E/I homoeostasis. Therefore, subsequent confocal analysis focused on quantifying immunopositive GAD+ profiles on MAP2+ neurons in different layers of the SMA motor CRTX compared to WT controls (Fig. 4e). Results showed a reduction of these profiles in layers 2/3 (−32% ± 9.31, *p* < 0.001) and 5 (-28% ±7.5, *p* < 0.001) in SMA motor CRTX (Fig. 4e–g). Next, to further assess changes in the overall inhibitory synapse puncta density in SMA samples compared to controls, we also immunostained the inhibitory postsynaptic marker gephyrin together with the presynaptic marker GAD to quantify their density on MAP+ somas (Fig. 4h). Results showed a significant decrease in the amount of gephyrin puncta contacting soma between WT and SMA brains both in layers 2/3 and layer 5 (-49% and -40%, respectively, Fig. 4i, j), as well as significantly reduced inhibitory synapse puncta density specifically in SMA in layer 5 of motor CRTX versus WT (−50%, Fig. 4l). Together, our results show that inhibitory synapses are largely affected in SMA motor CRTX, especially in the pyramidal layer 5.

Interestingly, similar alterations were also detected in a simplified in vitro system. Indeed. in primary neuronal cultures derived from SMA and control mice at a stage corresponding to their functional maturation (*DIV 15*) [54], we found an approximately 36% reduction in inhibitory synaptic complexes compared to controls in SMA neurons (Fig. S5), consistent with the in vivo findings. This decrease was accompanied by a trend toward reduced GAD+ presynaptic terminals and a significant reduction in gephyrin-positive postsynaptic puncta (-23%), indicating that these synaptic alterations are at least partially associated with intrinsic alterations of neuronal phenotype affecting GABAergic signalling.

**Fig. 5:**
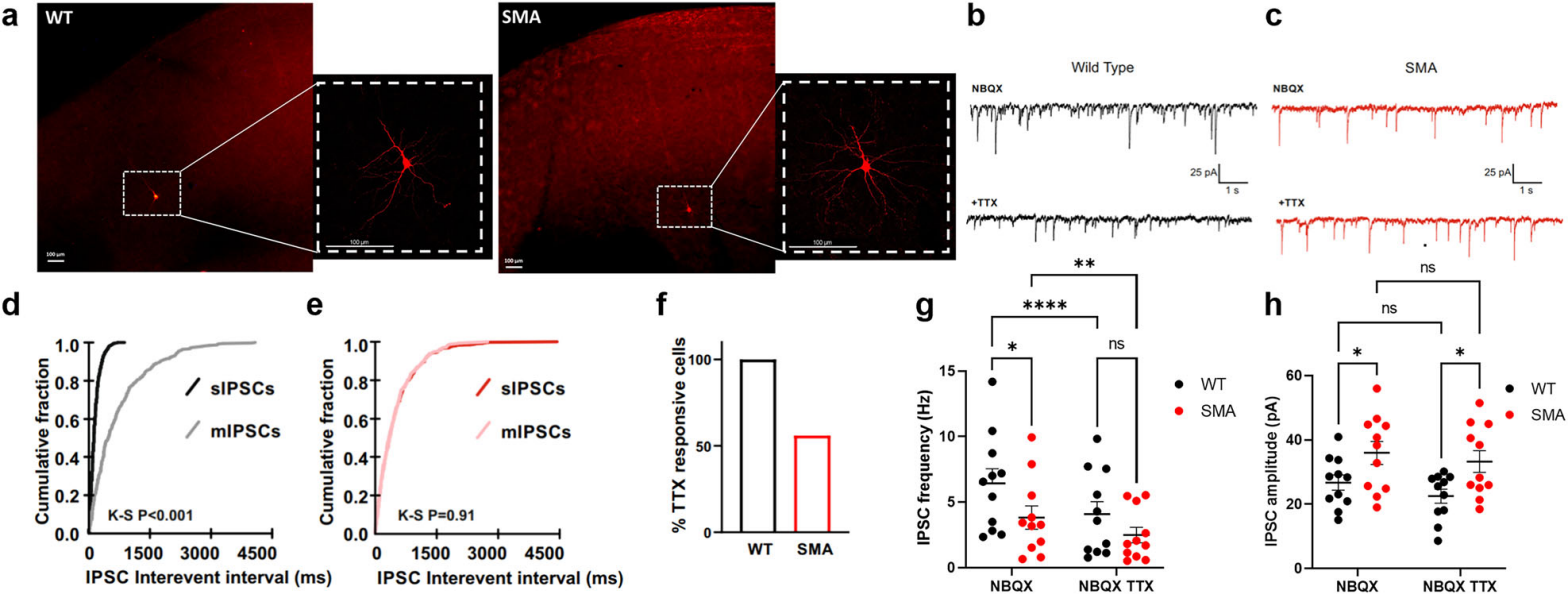
GABAergic activity-dependent inhibitory neurotransmission is affected in SMA motor CRTX. **a** Representative immunofluorescence images of biocytin-labelled motor cortex pyramidal neurons. Scale bar: 100 um. **b** Representative recording of sIPSCs (above) and mIPSCs (below) from a pyramidal neuron of a WT mouse. **c**. Representative recording of sIPSCs (above) and mIPSCs (below) from a pyramidal neuron of a SMA mouse. Recordings were performed as in G. **d** Cumulative distribution of sIPSCs and mIPSCs interevent intervals (ms) from the neuron in (**b**). TTX induced a clear right-shift of the interevent interval distribution (Kolmogorov-Smirnov test, *p* < 0.001). **e** Cumulative distribution of sIPSCs and mIPSCs interevent intervals (ms) from the neuron in (**c**) In this case, the distributions of interevent intervals were not affected by TTX (Kolmogorov-Smirnov test, *p* < 0.91). **f** Percentage of pyramidal neurons in which TTX significantly affected interevent interval (and hence frequency) of inhibitory currents (significant if K-S with *p* < 0.01). In WT mice, TTX decreased IPSC frequency in 100% of the recorded neurons (9 out of 9). Conversely, in SMA mice TTX affected IPSC frequency in only 56% of the recorded neurons (6 out of 11). **g** Multiple comparisons of mean IPSC frequency across genotypes before and after TTX (each dot represents a single recorded cell. Two-way ANOVA followed by Fisher’s post-hoc test; *p < 0.05; **p < 0.01; ***p < 0.0001; WT/ SMA mice: *n* = 4). **h** Multiple comparisons of mean IPSC amplitude across genotypes before and after TTX (each dot represents a single recorded cell. Two-way ANOVA followed by Fisher’s post-hoc test; *p < 0.05; **p < 0.01; ****p < 0.0001, ns: not significant; WT/ SMA mice: *n* = 4).

To test the functional impact of impaired GABAergic synapses in the motor cortex of SMA mice, we recorded action-potential-dependent (sIPSCs) and independent (mIPSCs) inhibitory synaptic transmission at visually identified layer 5 pyramidal neurons. Their morphology and localization were confirmed with intracellular injection of biotin (Fig. 5a). Frequency and amplitude of sIPSCs and mIPSCs from both WT and SMA mice are reported in Table 1. TTX treatment to block action potentials significantly reduced both frequency and amplitude of inhibitory events in neurons from WT mice, while it only slightly reduced frequency and had no effect on amplitude in SMA mice (Fig. 5b, c). Indeed, a significant reduction of GABAergic event frequency induced by TTX was observed in all (100%) of pyramidal neurons from WT mice (Fig. 5d–f), but only in 56% of neurons from SMA mice, indicating a reduced contribution of action potential-driven inhibition at inhibitory synapses of these animals. By comparing IPSC data before and after TTX treatment across mice groups, it emerged that the frequency of sISPCs, but not that of mIPSCs, was significantly reduced in SMA compared to WT mice (two-way ANOVA; Fig. 5g). Conversely, both sIPSC and mIPSC amplitudes were increased in SMA mice compared to WT mice (two-way ANOVA; Fig. 5h). Collectively, these data confirm an overall disruption of activity-dependent inhibitory transmission onto pyramidal neurons in SMA mice, but at the same time unveil a local TTX-resistant potentiation of inhibitory currents. Overall, both morphological and functional results highlight an E/I imbalance due to GABAergic impairment in the SMA motor cortex.

**Table 1:** Mean IPSC frequency and amplitude values (±SEM) from motor cortex pyramidal neurons in WT and SMA mice.

Mice	sIPSC frequency (Hz)	mIPSC frequency (Hz)	sIPSC amplitude (pA)	mISPC amplitude (pA)	n
WT	6.43 ± 1.11	4.07 ± 0.59***	26.72 ± 2.36	22.52 ± 2.17*	11
SMA	3.82 ± 0.88	2.49 ± 0.59*	35.96 ± 3.57	33.27 ± 3.38*	11

*P < 0.05, ***P < 0.001, paired t-test.

### SMN deficiency is correlated to neuronal GABAergic signalling and metabolism dysregulations

**Fig. 6:**
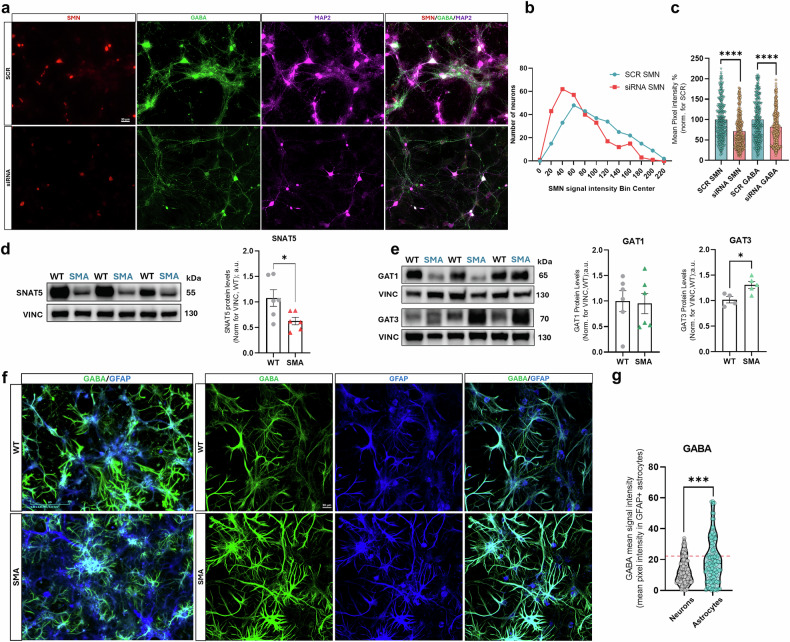
SMN deficiency is upstream to neuronal GABAergic signal and metabolism impairment in motor cortical areas. **a** Representative IF images showing the RNAi decreased SMN expression (red) and related GABA signal intensity (green) in siRNA primary cortical neurons (labelled from MAP2, magenta) from WT mice compared to SCR controls. Scale bar: 30 um. The results of SMN silencing in *DIV15* cortical neurons are then shown in (**b**) descriptive plot of the distribution of the number of siRNA and SCR neurons over a heterogeneous range of SMN signal intensity values. **c** Fold change histograms showing the average signal intensity values of GABA and SMN in ratio with those of the SCR neurons: the individual points show distributional differences of the neurons among samples (data are expressed as mean % ± SEM and normalized to SCR mean, referred as control; Student’s t-test, ****p < 0.0001;at least 100 neurons for each culture were analysed from *n* = 3 mice primary neuron cultures per group). Representative densitometries and protein levels quantifications of SNAT5 (**d**) and of the transporters GAT1 and GAT3 (**e**) in SMA P12 SM CRTX samples compared to WT controls [Data are expressed as mean ± SEM and normalized to VINC (referred as loading control) and to WT mean. Student’s *t*-test, *p < 0.05; for SNAT5 analysis: WT and SMA mice: *n* = 6; for GAT1 analysis: WT mice: *n* = 6; SMA mice: *n* = 6; for GAT3 analysis: WT mice: *n* = 4; SMA mice: *n* = 5]. **f** Representative widefield (left) and confocal (right) immunofluorescence images of WT and SMA primary cortical astrocytes (labelled by anti-GFAP antibody, in blue) cultures. Scale bar: 200 and 30 µm. **g** Quantification of GABA mean intensity in GFAP+ astrocytes among *DIV15* WT and SMA cortical cells. Red dashed line in the graph indicates WT relative GABA mean signal intensity. Analyses included at least 100 cells from six independent cultures for each group. Each dot on the graph represents an individual cell (Mann-Whitney test, ***p < 0.005; WT and SMA mice: *n* = 6 primary cell co-cultures).

Based on our findings in SMA SM CRTX, we investigated whether and how the observed dysregulation of GABAergic signalling and metabolism could be related to SMN deficiency. Given the role of SMN in RNA metabolism and actin dynamics [55], we aimed to assess whether SMN lack could cause upstream GABA neuronal dysfunction by affecting: i) neurodevelopment (e.g. cortical migration of GABAergic neurons) and/or ii) cellular GABA synthesis in the Glu/GABA/Gln (GGG) cycle and the GABA release. To investigate the expression of the genes involved in development and/or function of GABAergic neurons, we reanalysed the in vivo data sets “Transcriptome and translatome profiling from brains of a mouse model of severe SMA” (GEO: GSE102204) from the work of [55]. In this study, the authors assessed by RNA sequencing the global consequences of SMN deficiency on gene expression, at the transcriptional and post transcriptional levels, in tissue samples harvested from “Taiwanese” SMA mice [55]. In particular, we re-interrogated brain samples obtained from late-symptomatic (P7) animals, not only because this stage is phenotypically comparable with P12 SMA but also because most GABAergic neurons reach their assigned cortical laminar position around P7. We found no remarkable dysregulations in GABAergic IN markers and differentiation genes (FDR > 0.2) (Fig. S6a). Similarly, a gene set enrichment analysis (GSEA) performed on the same dataset, which could reveal subtler alterations, did not show significant skewing in the rank distribution of GABAergic interneuron markers (*p* = 0.187) and differentiation genes (*p* = 0.884) (Fig. S6b). These results argue against the possibility that SMN deficiency leads to GABA deficiency by altering the balance of cortical cell populations, suggesting the direct involvement of neuronal cell-specific mechanisms. To directly address this possibility, we evaluated the impact of decreasing SMN expression in WT primary cortical neurons by RNAi, compared to siRNA negative controls (SCR). Then, immunostaining analysis was performed in MAP2+ neuronal somas and the relative intensity of SMN and GABA fluorescent signal was quantified (Fig. 6a). Despite variability, the silencing efficiency was proved: SMN-silenced neurons (siRNA SMN) had a lower mean SMN signal intensity (mean SMN signal intensity: - siRNA neurons: 70.56% ± 2.42; -SCR neurons: 98.06% ± 2.83), due to an average SMN silencing efficiency of ~28% (SMN signal intensity in siRNA neurons: −28.51% ± 3.77, *p* < 0.0001) (Fig. 6b, c). In parallel, siRNA neurons showed a significant decrease in the average GABA signal intensity of ~18% (−17.88% ± 3.94, *p* < 0.0001) suggesting a correlation between reductions in SMN levels and GABA signal intensity (Fig. 6c). Notably, quantification in selected high-magnification fields revealed no differences in the proportion of GABA⁺ neurons among SMN-silenced neurons (siRNA SMN), SCR and untreated (NT) groups (~40% of total neurons) (Fig. S1d), indicating that the observed reduction in GABA signal is not attributable to general toxicity or loss of GABAergic neuron. Further analyses of neuronal fluorescence signal variance confirmed that GABA signal intensity tends to increase together with SMN intensity in both SCR- and siRNA-treated cortical neurons, with a moderate but significant correlation (Pearson r: SCR neurons: 0.48; siRNA neurons: 0.45; *p* < 0.0001) (Fig. S7). Overall, the above results indicate that neuronal SMN expression correlates with neuronal GABA signal intensity, highlighting the impact of SMN deficiency on GABA signalling dysregulation at neuron level.

Based on these findings, we explored whether the absence of SMN could directly affect genes involved in regulating GABA synthesis and release. In 2008 Zhang et al. [56] identified 73 genes with potential splicing pattern changes associated with SMN deficiency, including *Slc38a5*: the protein encoded by this gene is a system N sodium-coupled amino acid transporter SLC38A5/SNAT5, which provides net influx or efflux of Gln from the astrocytes to neurons, supplying it as a precursor for neurotransmitters in Glutamatergic and GABAergic neurons [40]. Here, we confirmed by WB analysis a significant reduction in protein levels of SNAT5 (-45.11% ± 17.88, *p* < 0.05) in the SMA P12 SM CRTX samples (Fig. 6d).

Such observations could explain the increased total cortical Gln levels observed (Fig. 2c), as reduced astrocytic SNAT5 expression likely impairs Gln efflux and causes intracellular Gln retention within astrocytes, thereby contributing to the overall tissue Gln accumulation. This mechanism could also account for the selective vulnerability of the SMA cortex to GABAergic synthesis impairment, further supporting a role for SMN deficiency in the negative regulation of the neuron–astrocyte glutamate–glutamine–GABA (GGG) cycle.

To verify this, we assessed the possible alteration of neuron-astrocyte GABA release and uptake in SMA CRTX by analysing the expression profile of GABA transporters (GATs). WB analysis of neuronal and astrocytic GAT (GAT1 and GAT3, respectively) expression showed no significant differences in GAT1 expression among samples, but a significant increase in GAT3 protein levels (+29.05% ± 9.37, *p* < 0.05) in SMA mice compared to controls (Fig. 6e). Our analysis at early symptomatic stage (P5) show that SNAT5 protein levels are already significantly reduced (−29% ± 7.487) in SMA mice, consistent with the hypothesis that its downregulation is an early event, likely related to SMN’s role in modulating SNAT5 splicing. By contrast, GAT3 protein levels do not differ between SMA and control mice at this stage, suggesting that GAT3 alterations may emerge later, possibly as part of disease progression (Fig. S2c)

These findings not only suggest dysregulation in GABA transport but also reinforce the possible role of astrocytes in the sequestration of GABA and its precursors, likely influencing neuronal levels of the neurotransmitter and the imbalance in SMA SM CRTX function in the late disease stage. To confirm such hypothesis, we evaluated the GABA signal intensity in co-cultures of primary SMA neurons and astrocytes (Fig. 6f): the GABA content of astrocytes was significantly higher in SMA astrocytes (23.29 ± 2.65, *p* < 0.005) compared to SMA neurons (14.45 ± 1.31) (Fig. 6g), while not significant difference occurred between WT neuron and astrocytes (Fig. S8), further showing an unbalanced distribution of the neurotransmitter among neural cells at SMA cortical level.

### Nusinersen-mediated SMN upregulation modulates GABAergic signalling and protein expression in SMA cortical cultures

**Fig. 7:**
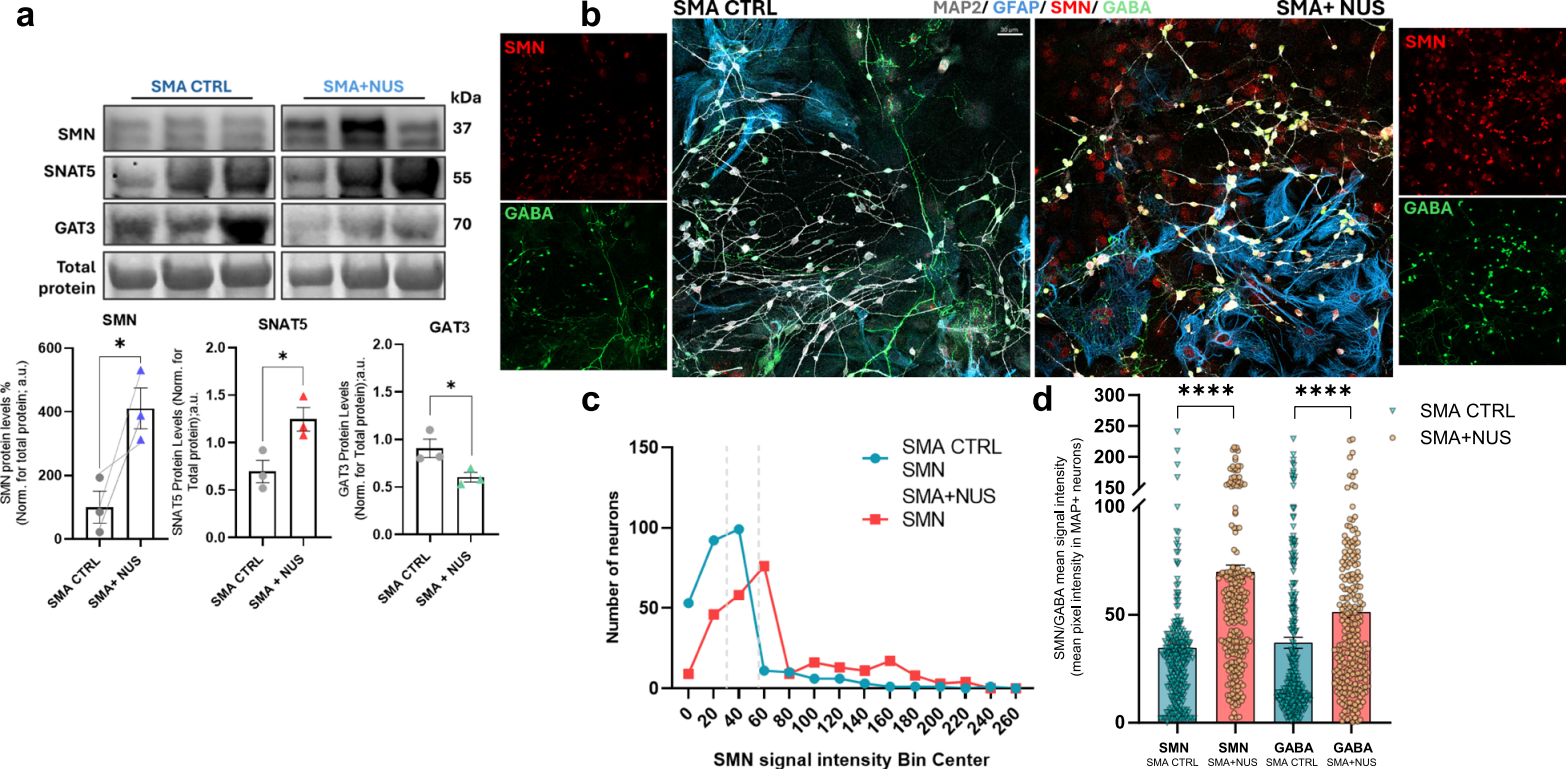
Effect of Nusinersen-induced SMN upregulation on GABAergic transporter expression and GABA signal levels in SMA cortical cultures. **a** Representative densitometries and protein levels quantifications of SMN and of the transporters SNAT5 and GAT3 in SMA primary cortical neurons and astrocytes co-cultures samples treated with Nusinersen (NUS) compared to relative untreated SMA controls (CTRL) [Data are expressed as mean ± SEM and normalized to total protein control (referred as loading control). Student’s *t*-test, *p < 0.05; for all the analysis: SMA CTRL and SMA + NUS samples: *n* = 3]. **b** Representative IF images showing the NUS increased SMN expression (red) and related GABA signal intensity (green) in NUS-treated SMA primary cortical neurons (labelled from MAP2, grey) compared to SMA CTRLs. Scale bar: 30 um. The results of SMN rescuing in *DIV15* cortical neurons are then shown in the **c**. descriptive plot of the distribution of the number of SMA + NUS and SMA CTRL neurons over a heterogeneous range of SMN signal intensity values. **d** Fold change histograms showing the average signal intensity values of SMA + NUS GABA and SMN in ratio with those of the SMA + CTRL neurons: the individual points show distributional differences of the neurons among samples (data are expressed as mean ± SEM; Mann–Whitney test, ****p < 0.0001; analyses included at least 100 cells from each culture for each group; *n* = 3 mice primary neuro*n* cultures per group. Each dot on the graph represents an individual cell).

To further evaluate the role of SMN deficiency in the dysregulation of GABAergic signalling at the neuronal level, we investigated whether restoring SMN levels could modulate the expression of genes and proteins involved in GABA synthesis and transport in cortical cultures. As a proof-of-concept experiment, primary cortical cultures were chronically treated with Nusinersen (NUS) (200 nM), an antisense oligonucleotide that modulates SMN2 splicing and is one of the currently FDA-approved drugs used as therapy for SMA [20, 57], from *DIV7* to *DIV15*, a period corresponding to neuronal functional maturation in vitro [54] **(**Fig.7) which is characterized by significant synaptic development and maturation in cortical cultures.

Among the experiments performed, we selected the cultures in which NUS induced at least a 1-fold increase in SMN expression for downstream analyses **(**Fig.7a, c). Immunoblotting analysis confirmed that SMN upregulation was accompanied by a significant increase in SNAT5 protein levels (+79% ± 17.13) and a reduction in GAT3 expression (−33% ± 10.92) compared to relative untreated SMA controls (CTRLs) **(**Fig.7a). Immunofluorescence assays further revealed a marked increase in GABA signal intensity ( + 38% ± 360.1) associated with elevated neuronal SMN (~1-fold increase) in SMA + NUS samples compared to SMA CTRLs **(**Fig.7b–d). These results suggest that Nusinersen-mediated SMN restoration modulates key components of the glutamate–GABA cycle, supporting the SMN-dependent amelioration of GABAergic metabolism and neurotransmission mechanisms.

**Fig. 8:**
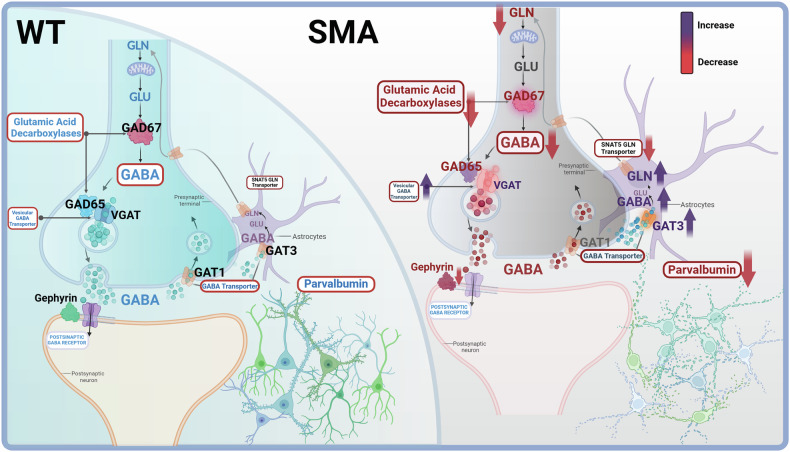
Neuronal GABA signalling and metabolic dysregulation in SM CRTX of SMA mice. Schematic cartoon representing dysregulation of GABA signalling and of metabolic pathway components at the neuronal level in the SM cortex of SMA mice (right) compared to WT controls (left). In brief, from top: In SMA, GABA precursor levels (GLN) are not supplied from astrocytes to SMA cortical neurons due to a significant decrease in SNAT5 transport, whose expression is proportionally correlated to the SMN deficiency. Moreover, GAD synthetic enzyme levels are significantly reduced, and the following lower GABA synthesis leads to dysregulation in GABA vesicular packaging and release (mediated by VGAT) and astrocytic GABA transport (mediated by GAT3, leading to astrocytic GABA accumulation). Downstream, SMA neurons show a lower number of GABAergic inhibitory synapses and a lower IPSC frequency, together with a reduction and morphological alteration of PV+ INs (Image created with BioRender.com).

According to the overall results, here we propose a mechanistic model underlying SMN deficiency-induced GABAergic pathway dysregulation in the SMA cortical motor network (Fig. 8).

## Discussion

Our study underpins an association between SMN deficiency and the neuronal dysregulation of GABAergic signalling and metabolism in the SM cortex of severe SMA mice, possibly indicating an alteration in the E/I balance in the cortical motor network, an aspect that still deserves further investigation. We further highlighted a SMN's pivotal role in influencing neuron-astrocyte interactions in GABA release and reuptake.

### GABAergic dysregulations contribute to shape UMN vulnerability in SMA mice SM CRTX

Our previous studies in the severe SMA mice model highlighted that reduced SMN protein levels result in a striking vulnerability of both LMN and UMN pools [19]. Concerning LMNs, SMN deficiency is known to trigger different cell death molecular cascades, such as apoptosis and autophagy, contributing to LMN degeneration [38, 39]. Moreover, also altered intrinsic excitability, synaptic connections, and chemical microenvironment appear to be at play in the LMN dysfunctions: herein, SMN loss has been closely linked to the impairment of excitatory afferent synapses in spinal sensory-motor circuits and increased Na+ channel activity, leading to LMN hyperexcitability and altered neurotransmission [32–34]. Consistently, recent evidence demonstrated that, beyond motoneuron hyperexcitability, spinal circuits in SMA undergo a maladaptive increase in inhibitory synaptic drive, resulting in a disrupted excitation/inhibition balance that further compromises motor output [35]. At the cortical level, several cellular processes are known to be involved in UMN death, such as excitotoxicity, disrupted spinal circuits, inflammation, oxidative stress, mitochondrial dysfunction, and protein aggregation [58, 59].

Nevertheless, regarding SMA UMNs, several info are still missing: we know that they undergo time-dependent degeneration, similar to LMNs, and have reduced spontaneous excitatory synaptic activity; however, the upstream mechanism of such cellular and functional impairment has been poorly characterized [10, 19]. To clarify these observations, here, we report a functional decline of GABA INs in the SM cortex of SMA mice assessed by reduction in the number and signalling of GABAergic neurons, changes in the expression and distribution of the GABA-synthesizing enzymes (GAD) and its precursor, Gln, as well as alterations in the density and morphology of PV+ INs. This was shown to result in a loss of GABAergic synapses and disruption of activity-dependent inhibitory neurotransmission. At the molecular level, reduced neuronal GABA can lead to cellular stress, including excitotoxicity, oxidative damage, and mitochondrial dysfunction, all of which are strongly associated with disease-specific neuronal degeneration [60, 61]. Thereby, we identified changes in motor cortical inhibition as a possible contributing factor in shaping SMA UMN vulnerability. These GABAergic dysregulations in SMA mice motor cortical areas converge to some extent with those assessed in neurodegenerative movement disorders. GABAergic dysfunction in the motor cortex of PD, HD and ALS is marked by the loss of IN inputs onto pyramidal cells, reduced intracortical inhibition, and an inability to modulate or enhance inhibition [1–6]. This dysfunction correlates with disease severity and contributes to dysregulated cortical activity that precedes motor output failure. In the SMA condition, we observe that UMNs degenerate over time, in parallel and not before LMNs [19]. However, late dysfunction of the UMN, potentially due to impaired neurotransmission, may in turn affect the excitability in the LMN spared by SMA progression, accelerating motor circuit disruption and worsening symptoms such as muscle weakness. In ALS, alterations in UMN excitation or inhibition have been reported to influence the LMN functions: changes in intracortical E/I balance within the motor cortex, as indicated by altered parameters like motor-evoked potential amplitude, cortical silent period, and central motor conduction time, likely disrupt corticospinal pathways, ultimately impacting LMNs, responsible for muscle control [62]. Conversely, Fletcher et al. [35] demonstrated that SMA spinal motor circuits exhibit a maladaptive enhancement of inhibitory synaptic input, resulting in excessive inhibitory tone and impaired motor output. Although apparently opposite to our cortical findings of reduced inhibitory regulation, these data likely reflect distinct manifestations of a shared SMN-dependent vulnerability of inhibitory networks. Such level-specific alterations in E/I balance along the cortico-spinal axis may represent compensatory or maladaptive responses that ultimately converge to impair motor circuit integrity and contribute to disease progression. Examining cortical inhibition could be another key aspect in the aetiology of SMA disease and in understanding the mechanisms underlying the progression of SMA neurodegeneration.

### Deterioration of the GAD67-PV + IN pathway impairs GABAergic neurotransmission in the late disease stage in SMA mice SM CRTX

There is evidence that impairment of the GAD67-PV + IN pathway causes long-lasting deficits in neuronal network function. Rodent models with reduced GAD67 exhibit a significant decrease in neuronal GABA content and release in PV+ INs, which consequently show structural and functional impairments, including reduced axon branching and defects in perisomatic synapse formation. As a result, these models also show altered synaptic properties, pyramidal neuron disinhibition, and impaired locomotor coordination, underscoring the critical role of the GAD67-PV + IN pathway in GABAergic transmission and motor control [31, 42, 43, 63–65]. Consistent with this evidence, our immunohistochemical and molecular findings reported both a significant reduction in GAD67 enzyme expression and neuronal distribution, along with a striking reduction and altered morphology of PV+ INs in SMA SM CRTX mice. Furthermore, we assessed an overall decrease across cortical layers 2/3 and 5, reaching significance in layer 5, in SMA SM CRTX of GABA+ neurons and, together with a concomitant significant reduction of both PV+ INs and GABAergic presynaptic profiles (GAD+), consistent with a selective vulnerability of output-layer circuits rather than pan-cortical degeneration. Nevertheless, we observed a specific and significant reduction of PV+ INs and inhibitory synapse complexes in motor cortical layer 5, paralleled by a reduced frequency of spontaneous GABAergic events at pyramidal neurons, adding compelling weight to the assessment of UMN SMA disinhibition. Consistent with the ex vivo findings, our analysis of inhibitory synapses in primary neuronal cultures derived from SMA and control mice indicates that these synaptic alterations are at least partially cell-autonomous and likely reflect an intrinsic neuronal impairment of GABAergic signalling. Additionally, our functional results in SMA mice indicate that alterations in IN properties mainly affect action-potential-dependent GABAergic transmission rather than TTX-resistant spontaneous GABA release, suggesting a major impairment of the machinery directly involved in evoked GABA release [66, 67] or in the availability of vesicle pools for evoked neurotransmission [68]. A reduction of spontaneous action potential-dependent inhibitory transmission has been described in other neurological disorders where E/I balance is altered (for instance, in epilepsy due to irradiation induced cortical dysplasia and heterotopy [69]). Here, this reduction was accompanied by a significant increase in the amplitude of inhibitory events. In congenital neurological diseases, where inhibitory circuits are constitutively altered, the potentiation of inhibitory currents at the postsynaptic site may likely represent a compensatory strategy known as homoeostatic plasticity [70–72].In keeping with this, we also found that the vesicular GABA transport protein (VGAT) was increased, thus allowing more GABA to be packed into the inhibitory synaptic vesicles.

VGAT is expressed in both GABAergic and glycinergic terminals and can transport β-alanine, suggesting that changes in its levels may affect multiple inhibitory neurotransmitters [50, 73, 74]. In this study, we focused on GABAergic markers and did not assess glycinergic components. Importantly, even moderate VGAT alterations, as detected in SMA mice with a modest but significant increase at both the early symptomatic and late disease stages, can significantly affect inhibitory synaptic currents and physiological responses [75].

These functional adaptations, while attempting to potentiate inhibition in a context where inhibitory transmission is impaired, are unlikely to have a relevant impact on the function of cortical circuits. As shown in other models in which inhibitory neurons are reduced or non-functional, homoeostatic compensation may partly rebalance inhibition and excitation, but cannot fully normalize neural function [71]. Indeed, the functional role of inhibition is not solely determined by its strength, but also by the timing relative to the excitatory drive [76, 77]. Feedforward inhibition, which depends on reliable interneuron spiking, is essential for sharpening the temporal window of excitation and ensuring coherent integration of sensory inputs into motor circuits. Thus, the concomitant potentiation of inhibitory currents alone is unlikely to efficiently control pyramidal function in a timely manner and is consistent with the sensory input received.

A marked reduction in GAD67 expression was also reported in the primary motor cortex of ALS SOD1 mice during the late presymptomatic phase (8.5–10 weeks). Herein, since at this stage of the disease PV+ INs in layer 5 were found hypoactive, showing reduced GAD67 expression, it has been hypothesized that the decline in GAD67 mirrors dysregulation of GABA synthesis due to reduced PV + IN activity and consequent increased pyramidal cell activity [41, 78]. In SMA SM CRTX, the reduction of GAD67, accompanied by morphological and density alteration in PV+ INs in advanced disease, may reflect an impairment of PV + IN activity, triggering GABAergic dysfunction and UMN disinhibition. Compared to the SOD1 mouse model, severe SMA mice have a dramatically shortened lifespan (~13 days), starting early symptomatic at P5 and becoming fully symptomatic at P10. PV+ interneurons in mice originate from the medial ganglionic eminence (MGE) and migrate into the cerebral cortex, where they differentiate and begin expressing parvalbumin, which is not reliably detected before ~P12 [79, 80]. During this period, cortical GABAergic synapses are still undergoing the excitatory-to-inhibitory switch, which occurs mainly between P7 and P14 [10, 45–47, 49]. Our analyses at P12, corresponding to the late disease stage in severe SMA mice, fall within this window, when most neurons have completed the switch to inhibitory function, whereas at earlier ages (≤P7–P10) GABA is still partially depolarizing, complicating interpretation of inhibitory neuron reductions.

Therefore, in the SMA murine model, it is challenging to pinpoint the exact timing of potential PV + IN hypoactivity before the fully symptomatic stage. No changes in GAD65 or GAD67 protein levels were observed at P5, indicating that the deficits detected at P12 emerge after the GABA switch and are not due to early developmental defects. This suggests that the observed alterations in the GAD67–PV⁺ interneuron pathway are not caused by impaired migration or early differentiation of GABAergic neurons but likely arise from post-developmental neurometabolic disturbances. Aiming to in-depth trace the origin of the SMA cortical alteration in the GAD67-PV + IN pathway, through bioinformatic analysis, we excluded the possible neurodevelopmental effect induced by SMN deficiency on the differential expression of GAD and on the migration and development of GABAergic neurons and INs. Nevertheless, according to our results, we speculated that impaired GABA synthesis in PV+ INs might depend on neurometabolic issues. Our neurotransmitter profiling in the whole cortex of severe SMA mice showed that both GABA and Glu levels remained unchanged compared with WT controls, while L-Gln levels were increased, leading to a higher L-Gln/L-Glu ratio at the late disease stage. Although these results might appear contradictory, they can be reconciled by our histological findings showing a reduction of intracellular/somatic GABA signal at P12, particularly in layer 5, consistent with the selective vulnerability of output-layer circuits rather than pan-cortical degeneration, as GABAergic neuron numbers were relatively maintained across other layers. Overall, our data support a model in which GABAergic dysfunction in SMA is primarily functional and regionally selective, rather than reflecting global neurotransmitter depletion. These results exclude a simple depletion of the tissue GABA pool and suggest that localized neuronal deficits may be masked by compensatory changes in other cortical regions or cell types. Notably, the cortical homogenates used for HPLC also include glial components, and the reduction of GABAergic signal in the vulnerable neuronal population is likely counterbalanced by glial contributions to the total cortical pool. The apparent uncoupling between elevated cortical Gln levels and unchanged total GABA content may reflect impaired astrocyte-to-neuron glutamine transfer and reduced neuronal conversion efficiency, leading to intracellular Gln accumulation and inefficient neuronal GABA synthesis. Indeed, in the GGG cycle, Gln flows from astrocytes to neurons, where it is converted into Glu and subsequently into GABA by GAD enzymes; Glu and GABA then flow in the opposite direction. The increase in the L-Gln/L-Glu ratio in late symptomatic phases could reflect a failure in neuronal conversion of L-Gln into L-Glu at this stage, affecting neuronal GAD-mediated GABA synthesis. A study by Qureshi et al. [81], focusing on the transporter Slc38a1/SNAT1 for Gln uptake into nerve terminals in the GGG cycle, highlighted that GABA synthesis in GABAergic INs, particularly PV+ INs, strongly depends on astroglia-derived Gln [81]. This evidence leads us to hypothesize that, in the cortical motor areas of SMA mice, dysregulations in PV + IN GABA synthesis trigger a disruption of inhibitory neurotransmission in the late disease stage, which occurs simultaneously and in response to late-stage impairment of Gln neuron-astrocyte transport and/or metabolism.

### SMN deficiency disrupts neuron-astrocyte interaction in GABAergic neurotransmission

Next, we assessed the role of SMN deficiency in triggering the observed SMA GABAergic neuronal dysfunctions. The in vitro analysis showed that SMN levels correlate with the intensity of cortical neuronal GABA signalling. Since alterations in GABAergic IN pathways often result from impaired genetic factors affecting RNA metabolism (e.g., TDP43 in ALS) [82], we focused on the contribution of SMN as an RNA-binding protein in these dysregulations in SMA. SMN deficiency causes profound changes in RNA metabolism by altering the snRNA repertoire and disrupting pre-mRNA splicing [55, 56]. Hence, we assessed the potential changes in the splicing pattern of brain genes associated with SMN deficiency from exon array analysis performed by [56], specifically focusing on those involved in the GABA synthesis and release pathway. We noticed, among the most affected genes, SLC38A5/SNAT5, which encodes a transporter protein that ensures the net influx of astroglia-derived Gln to Glutamatergic and GABAergic neurons [83].

Our results confirmed that in SM CRTX, SNAT5 expression is strongly reduced in SMA mice SM CRTX. Therefore, we propose a model in which SMN deficiency perturbs neuronal GABAergic metabolism and signalling in PV+ INs, triggering abnormal L-Gln retention in astrocytes, due to the induced failure of the Gln transporter. Astrocytes, through Na + -dependent pathways, influence neuronal GABA metabolic supply via SNAT3-mediated GLN supply, but are also upstream of neuronal-released GABA uptake via the GAT3 transporter, as it is required for metabolic processes. Therefore, GAT3 overexpression could dramatically reduce extracellular GABA, shifting the E/I balance toward increased neuronal excitation [84]. Our results revealed both a significant increase in astrocytic GABA transporter GAT3 expression in the SM cortex of SMA mice and GABA signal intensity in in vitro SMA cortical astrocytes compared to neurons. Therefore, we attributed GAT3 overexpression in SMA SM CRTX to the increased metabolic demand for GABAergic uptake by astrocytes, following SMN deficiency-induced GGG cycle disruption (due to SNAT5 downregulation) in the neuron-astrocyte network. This SMN-dependent pathogenic mechanism could also contribute for amplifying astrocyte metabolic dysfunctions and reactivity, as also suggested in other neurodegenerative conditions [85], adding new insight to our previous assessment of a marked astrogliosis paralleling the loss of motor cortical layer 5 pyramidal cells in late-stage SMA mice [19]. Importantly, SNAT5 protein levels are already significantly reduced at the early symptomatic stage, suggesting that its downregulation is an early event, likely linked to SMN’s role in modulating SNAT5 splicing. By contrast, GAT3 protein levels at P5 do not differ between SMA and control mice, consistent with the physiological immaturity of the GABAergic system at this stage: PV⁺ interneurons have not yet reached full maturation, GAD enzyme levels remain unchanged, and the developmental GABA switch from excitatory to inhibitory has not occurred. Together, these observations support the idea that GAT3 alterations are not a primary defect, but a downstream event emerging later in disease progression.

Our proof-of-concept experiments with Nusinersen-treated primary cortical neurons provide further support for the model of SMN-dependent dysregulation of GABAergic metabolism in SMA. Chronic exposure to Nusinersen restored SMN expression and led to a significant upregulation of SNAT5, along with a reduction in GAT3 and a concomitant increase in GABA levels. These findings suggest that SMN restoration can partially normalize the neuron-intrinsic components of the GGG cycle. Potentially, by rescuing SNAT5 expression, the Nusinersen-induced increase in SMN may enhance neuronal Gln supply and support GABA synthesis in PV+ interneurons. At the same time, the reduction in GAT3 could help rebalance extracellular neurotransmitter levels, potentially mitigating the excitation/inhibition (E/I) imbalance observed in the SMA motor cortex. Further studies could investigate whether similar effects occur in astrocytes, including reactive ones, to better understand how neuron-astrocyte interactions are influenced by SMN-targeted therapies.

### Limitations of the study

In this study, we focused only on the inhibitory dysfunction in shaping UMN vulnerability, although we cannot exclude the potential role of altered Glutamatergic metabolism and signalling in this process [86]. Furthermore, reactive astrocytes may exacerbate GABAergic neuronal dysfunction by impairing Glutamate uptake and causing hyperexcitability, or even overproduction of GABA (via GAD-independent pathways), leading to aberrant tonic GABA release [84, 86, 87]. Further analyses (as for tonic GABA release) are needed to clarify whether and which of these mechanisms occur in the motor cortical network of SMA mice. Although no significant cortical neurophysiological sex differences have been reported in the SMNΔ7 model [88], the lack of sex-specific analysis represents a limitation of the present study. Future investigations should address potential sex-dependent effects on cortical GABAergic alterations to achieve a more integrated and comprehensive understanding of neurometabolic changes in SMA and their contribution to disease mechanisms [89].

Finally, this study focused specifically on the sensorimotor cortex, due to its selective vulnerability in SMA. While other brain regions, including the cerebellum, have been implicated in motor function [10], we did not assess GABAergic pathways outside the sensorimotor cortex. Comparative analyses across multiple brain regions with layer-specific distributions assessments of inhibitory neurons (including also other inhibitory INs classes, e.g., SST+, CB+, VIP+) are important next steps, which could provide additional insight into regional vulnerability and the broader impact of SMN deficiency and should be considered in future studies.

### Therapeutic implications of the study

Our findings of impaired PV + IN density and function and disrupted GABAergic transmission in late-stage SMA mice provide a mechanistic framework to reinterpret existing human evidence. By temporally mapping these alterations to a post-maturational window (P12 in SMA mice), we highlight a phase in which functionally mature INs begin to fail, likely as a consequence of SMN deficiency–induced disruption of the neuron–astrocyte GGG cycle, while offering a plausible developmental origin for the cortical dysfunction observed in SMA. This timing resonates with neuroimaging and histopathological studies in patients, where phenotypic severity correlates with either cortical thinning or compensatory remodelling [11–17]. Such changes may reflect an early disruption of inhibitory circuit function—particularly involving PV+ INs —whose malfunction could contribute to impaired cortical output and maladaptive plasticity. These results support the hypothesis that altered GABAergic signalling is not merely a secondary consequence but may actively shape disease progression and cortical vulnerability, warranting further investigation in preclinical and clinical settings.

In this concern, administration of GABA chemical analogues and GABA targeting drugs (such as AEDs/APDs) in SMA case reports and clinical trials was shown to prevent motor function deterioration and attenuate disease progression in SMA patients [20, 23–27, 29]. While these studies did not generally report significant side effects, contradictory findings on muscle strength, motor function, and survival emphasize the need for further research on the long-term use of these drugs in SMA patients [20, 23–27, 29]. Her,e we add compelling weight to therapeutic treatments that augment inhibitory neurotransmission in SMA and offer insights into further approaches targeting motor cortical inhibitory INs. In this concern, Khademullah et al. [78] showed that chronic chemogenetic activation of hypoactive PV+ INs in motor cortical layer 5 rescued both UMN and LMN-related symptoms and delayed motor impairments in SOD1-ALS mice, suggesting potential for translatable treatment to ALS MND patients [78]. On this line, our results encourage further screening or repositioning of GABAergic IN-targeted drugs in SMA models, aimed at assessing the potential rescue of UMN dysfunction and subsequent delay of LMN degeneration, and thus locomotion impairment. Given the role of the SMN in regulating neurotransmitter metabolism and cycling, experimental validation of multidrug approaches that systematically restore the SMN (targeting UMN/LMN dysfunction), while enhancing cortical motor inhibitory neurotransmission, may offer a novel translational perspective to preserve corticospinal homoeostasis in SMA patients.

In conclusion, our study focused on unravelling the regulation of the GABAergic pathway in SMA, providing new evidence supporting the involvement of the cortical motor network in exacerbating disease progression through E/I imbalance, while also linking SMA to other neurodegenerative movement disorders. This, in turn, offers a bench-to-bedside strategy, advancing our understanding of the disease and addressing patient needs through improving ongoing therapies. Furthermore, exploring the SMN role in regulating molecular pathways involving neural cell crosstalk fills gaps in current research, paving the way for groundbreaking advances in SMA comprehension and treatment.

## Supplementary information

Supplementary Materials and Methods

Supplementary Figures 1 to 8

Full unedited immunoblotting membranes

## Data Availability

All data generated or analysed during this study are included in this published article [and its supplementary information files].
