# Supplementary material for: Changes in the cortical GABAergic inhibitory system in a Spinal Muscular Atrophy mouse model
Source: Cell Death Dis. 2026 Feb 28;17(1):285. doi: 10.1038/s41419-026-08520-8 (PMC13031913; doi:10.1038/s41419-026-08520-8)
Supplement: Supplementary file 1 — Supplementary Materials and Methods [file 41419_2026_8520_MOESM1_ESM.pdf]

Supplementary Materials and Methods for: "Changes in the cortical GABAergic inhibitory system in a Spinal Muscular Atrophy mouse model"

Giovanna Menduti[1,2, #], Francesco Ferrini[3,4], Anna Caretto[1,2], Amber Hassan[5,6], Raffaella di Vito[6,7], Giada Beltrando[1,2], Davide Marnetto[2], Alessandro Usiello[6,7], Ferdinando Di Cunto[1,2], Marina Boido[1,2, *] and Alessandro Vercelli[1,2,*]

* These authors contributed equally to this work

# Corresponding author: Giovanna Menduti; full postal address: Neuroscience Institute Cavalieri Ottolenghi, Orbassano, 10043 Turin, Italy; telephone: + 39 011 670 6613; email: giovanna.menduti@unito.it

Animals

Our studies were conducted using SMAΔ7 mice (from now SMA; stock no. 005025, Jackson Lab, Bar Harbor, ME, USA) as a murine model of severe SMA [1]. The breeding colony was maintained by interbreeding Smn+/–SMN2+/+SMNΔ7+/+ mice, animals had free access to food and water and were kept into regular cages under 12/12-h light/dark cycle. Animals of both sexes were used in this study; however, sex was not considered as an experimental variable, as no significant sex-dependent differences have been reported for this model [2]. For experimental procedures, the animals were genotyped at P0–1 by processing the DNA extracted from tail tissue samples [3] by PCR assays. Hence, genotyping protocol [according to [4]] assessed the presence of the two human transgenes (*SMN2* and *SMNΔ7*) and the three possible genotypic variants of the Smn locus mice (*Smn+/+, Smn+/– or Smn-/-*). Data were obtained from tissues harvested from SMA mice and WT controls, sacrificed at P1-P2

(pre-symptomatic stage), P5 (early symptomatic stage) and P12 (late symptomatic stage). All efforts were made to minimize the number of animals used and the suffering levels.

Animal sacrifice, tissue collection and processing

For immunohistochemical analysis, P12 mice (WT n = 6, SMA n = 6) were anesthetized by gaseous anaesthesia (3% isoflurane vaporized in O_2/N_2O 50:50) and perfused transcardially with 4% buffered PFA, pH 7.4. Then, whole brain was dissected, postfixed in 4% PFA for 2 h and incubated overnight in 30% sucrose in 0.1 M phosphate buffer solution. Next, samples were embedded and frozen in cryostat medium (Killik, Bio-Optica) and cut with a cryostat (Microm HM 550; Thermo Fisher Scientific) into coronal 40 µm thick free-floating sections, finally stored at 20 °C in an antifreeze solution (30% ethylene glycol, 30% glycerol, 10% PB; 189 mM NaH_2PO_4; 192.5 mM NaOH; pH 7.4). For both HPLC and immunoblotting analyses, animals were sacrificed by cervical dislocation at the following postnatal ages: P2 (for HPLC: WT n = 7, SMA n = 7), P5 (for HPLC: WT n = 7, SMA n = 7) and P12 (for HPLC: WT n = 7, SMA n = 7; for WB: WT n = 6, SMA n = 6). The brain was dissected to collect the whole CRTX or specific SM cortex samples, subsequently frozen in liquid nitrogen and then stored at −80°C. For *in vitro* neuron and astrocyte co-cultures, P1 mice (WT n = 6, SMA n = 6) were sacrificed by decapitation and the whole brain rapidly removed for dissection and cortical cell isolation. For electrophysiological experiments, an additional group of P12 mice (WT n = 4, SMA n = 4) was sacrificed with a lethal dose of sodium pentobarbital (30 mg/kg, intraperitoneal). and the brains were quickly removed and submerged in ice-cold, cutting solution containing (in mM) 252 sucrose, 2.5 KCl, 2 $MgCl_2$, 1.5 $CaCl_2$, 1.25 NaH_2PO_4, 26 $NaHCO_3$, 10 Glucose, 1 kynurenate and bubbled with 95% O_2/5% CO_2.

HPLC analysis in mice CRTX samples

Frozen mouse CRTX samples were pulverized and homogenated in liquid nitrogen. Trichloroacetic acid (TCA) 0.2 M was added at 10 µL/mg ratio, and samples were sonicated (4 cycles,

10 s each), and centrifuged at 13,000 × g for 20 min. TCA supernatants containing amino acids were then neutralized with NaOH, subjected to pre-column derivatization with o-phthaldialdehyde (OPA)/N-acetyl-L-cysteine (NAC), and resolved on a UHPLC Agilent 1290 Infinity (Agilent Technologies, Santa Clara, CA, USA) using a ZORBAX Eclipse Plus C8, 4.6 × 150 mm, 5 µm (Agilent Technologies, Santa Clara, CA, USA) under isocratic conditions (0.1 M sodium acetate buffer, pH 6.2, 1% tetrahydrofuran, and 1.5 mL/min flow rate). A washing step in 0.1 M sodium acetate buffer, 3% tetrahydrofuran, and 47% acetonitrile was performed after every single run. Identification and quantification of L-Glutamate (L-Glu), L-Glutamine (L-Gln), and GABA were based on retention times and peak areas, compared with those associated with external standards. All the precipitated protein pellets from mice samples were solubilized in 1% sodium dodecyl sulfate (SDS) solution and quantified by bicinchoninic acid (BCA) assay method (Pierce™ BCA Protein Assay Kits, (Thermofisher scientific, Rockford, IL, USA). The concentration of amino acids in CRTX homogenates was normalized to the total protein content and expressed as nmol/mg protein.

Isolation and co-culture of primary mouse neurons and astrocytes

Neocortices from P1 WT and SMA mouse pups were harvested and dissociated; cortical cells were isolated for co-culture of neurons and astrocytes by re-adapting related protocols [5,6] (a schematic overview of the experimental workflow for primary neuron–astrocyte co-cultures is shown in **Fig. S1 a-b**). Briefly, after brain dissection (in 100 mm petri dishes with culture media), the harvested samples from each mouse were separately incubated in a trypsin-DNAse solution (Sigma-Aldrich) (15 min, 37°C, 5% CO_2); next, the solution was removed and the dissociation media added, followed by tissue trituration 10–15 times with a larger and then smaller diameter fire-polished Pasteur pipette. After tissue chunks had settled, all the supernatant (containing dissociated neurons) was transferred into tubes containing fetal bovine serum (FBS) (Sigma-Aldrich), suitable for the following sedimentation of the cell pellet with centrifugation (1,000 rpm, 10 minutes, RT). Next, cell pellet was resuspended in plating medium and cortical cells were plated at a density of 150,000 cells/cm^2 on substrates

precoated with 0.5 mg/mL of poly-L-lysine (Sigma-Aldrich). Primary cortical cells were allowed to adhere for 4 h before the medium was changed to the co-culture medium. Half-media changes were performed every 3/4 days, then cells were cultured until *DIV 15*. WT co-cultures were used as a reference to assess the purity and reproducibility of the neuronal–astrocytic co-culture model. Quantification of neuron and astrocyte percentages in co-cultures was performed by manually counting $MAP2^+$ neurons and $GFAP^+$ astrocytes among at least 100 cells per culture from three independent WT co-cultures (Fig. S1b). Consistent with the adopted protocol, our cultures contained approximately 60% $MAP2^+$ neurons and 40% $GFAP^+$ astrocytes, as expected for a mixed neuron–astrocyte population. For subsequent immunocytochemical assay, cells were washed with Dulbecco's Phosphate Buffered Saline (DPBS), then fixed with 4% paraformaldehyde (PFA) for 15 min, then the latter was removed and followed by three washes in PBS. Fixed cells were stored in a PBS and 0.02% sodium azide NaN 3 % solution at 4°C.

Immunofluorescence assays on brain sections and primary cortical cells

For immunohistochemistry (IHC) analysis, free floating brain sections underwent immunofluorescence staining, as previously reported in [7]. Coronal brain sections were cut on a cryostat at 40 µm thickness, except for immunostaining of biocytin-filled neurons (**Fig. 4M**), where slices were acutely cut on a vibratome at 300 µm thickness (see patch clamp methods) and subsequently fixed in PFA 4% for 30 min. After a 30 min permeabilization with 0.3% v/v Triton X-100 (ThermoFisher) solution in 0.01M PBS (PBS-T; pH 7.4), the samples were blocked with a solution of 10% v/v normal donkey serum (NDS) in PBS-T (blocking solution) for 1 hour and then incubated overnight at 4°C in different combinations of primary antibodies, and next with a mix composed by appropriate fluorophore-conjugated secondary antibodies, and with 4′, 6 Diamino-2 phenyindole Dilactate (DAPI), for 2 hours. For immunostaining of biocytin-filled neurons, coronal brain slices were incubated O/N at 4°C with PBS-T solution (at 0.1% v/v Triton X-100) plus conjugated antibody to reveal biocytin. Primary and secondary antibodies, with relative dilutions, are

listed in **Table S1**. Samples were washed and coverslips were mounted with anti-fade mounting medium Mowiol. For immunocytochemistry (ICC) analysis, the fixed cells were first washed twice with PBS, followed by a 15-min permeabilization in PBS-T with 0.25% v/v Triton X-100. Samples were incubated in blocking solution for 30 minutes. Thereafter, samples were incubated overnight at 4°C in different combinations of primary antibodies (**Table S1**) diluted in NDS 2% and PBS-T solution, and next, following three washing with PBS, with a mix composed by appropriate fluorophore-conjugated secondary antibodies (**Table S1**) in NDS 2% and PBS solution for two hours. Subsequently, the cell glass coverslips were washed 3 times with PBS and mounted on microscope slides with anti-fade mounting medium Mowiol.

Imaging and analysis

Image acquisition for immunofluorescence analysis in mice SM CRTX at P12 was performed with a Leica TCS SP5 laser scanning confocal microscope (Leica Microsystems), with a Zeiss Axioscan Z.1 digital slide scanning microscope (Carl Zeiss AG) and with a Zeiss Apotome 3 optical scanning microscope with structured illumination (Carl Zeiss AG). Confocal acquisitions were performed on optical sections scanned using a 40x oil objective (N.A. 1.30), throughout 6-µm section thickness, in z-steps of 0.5 µm, with a resolution of 1024 × 1024 pixels, frequency of 200 Hz or, for inhibitory GAD+ synapses imaging (**Fig. 4E**) by using a 40x oil objective with 2.5 X zoom, at 0.2 µm z-steps, with a resolution of 1024 × 1024 pixels, frequency of 100 Hz. Digital slide scanner microscope acquisitions were performed on optical sections scanned using a 20x objective (N.A. 0.8), throughout 15-µm section thickness, in z-steps of 3.5 µm, with a resolution of 130844 × 60842 pixels. Optical scanning microscope acquisition were performed in high-resolution optical sectioning on sections scanned using a 20x objective (N.A. 0.8) objective, throughout 6 µm section thickness at 0.5 µm z-steps, with a resolution of 1024 × 1024 pixels. Imaging analyses were performed on maximum

projections Z-stacks images or single optical planes by using FIJI software (ImageJ) [8], for each analysis the following are the methods, and parameters.

In quantitative analysis of GABAergic signal, the GABA immunostaining highlighted, as expected, the rich cortical GABAergic network (cell bodies and dendritic branches - which showed punctate staining) allowing subsequent analysis of neurotransmitter immunopositive profile densities, expressed as % of "particles", *i.e.* GABA immunopositive objects segmented into thresholded image areas. Therefore, confocal photomicrographs of SM CRTX were analysed segmenting the thresholded image (manually set threshold range; *Adjust Threshold tool*) with the *Analyze Particle* command, determining the particle size range for GABA+ object staining. Results are reported as particle area fraction (percentage of pixels in the image). Quantitative analysis of GABAergic signal at P12 included four WT and five SMA mice. For each mouse, at least four coronal sections were analysed, and a minimum of four confocal fields per section were examined in the M1 and S1 cortices. Quantification values were averaged across fields and sections to obtain a single mean value per animal. Violin plots show the distribution of these values across animals within each group, including median and variability. Concomitant analysis of the number of GABA+ cells was achieved by manually counting GABA+ neuronal somas in thresholded images. In details, GABA+ cells were manually counted in confocal images from defined cortical regions using consistent imaging and analysis parameters. Cell counting at P12 included six WT and six SMA mice. For each mouse, at least four coronal sections were analysed, and at least four confocal fields per section were counted in the M1 and S1 cortices. Counts were averaged across fields and sections to obtain a single mean value per animal.

Quantitative analysis of $GABA^+$ neurons in the SM cortex layers was performed on whole coronal brain section images acquired with a digital slide scanner microscope. Specifically, each coronal brain section was analysed on maximum intensity projections of 15 μm optical stacks (z steps 3.5 μm), largely capturing neuronal somata and minimizing counting errors from partial or

overlapping cells. To enable region-specific analysis, each coronal section was matched to the corresponding plates of the Allen Brain Atlas (https://mouse.brain-map.org/static/atlas). Selected atlas plates corresponding to the coronal slices analysed were resized and superimposed onto the brain sections using Adobe Illustrator, then converted into binary masks in FIJI and overlaid onto the sections to guide the identification of cortical areas of interest, including M1 and S1. Using these overlay masks, regions of interest (ROIs) were manually delineated on thresholded images. Cortical layers within each ROI were identified based on anatomical landmarks and further confirmed by DAPI staining, which highlights the typical laminar and columnar organization of neurons in M1 and the barrel field of S1. Within each layer-specific ROI, $GABA^+$ neuronal somata were manually counted and normalized to the number of $DAPI^+$ nuclei, quantified using the Watershed and Analyze Particles tools in FIJI. Counts were expressed relative to the total ROI area. This procedure ensured accurate quantification of $GABA^+$ neurons within defined cortical layers of M1 and S1 shown in **Figure 1d**. At least four coronal sections per animal were analysed, with three animals included per group. Counts were averaged across fields and sections to obtain a single mean value per animal. A similar workflow was applied for the quantification of GAD67 and PV immunopositive profile densities. In this analysis, the overlay masks derived from the reference atlas tables were used to manually delineate the regions of interest (M1 and S1 ROIs) on thresholded images. These regions were identified according to anatomical landmarks defined in the atlas and verified by DAPI staining (confirming the characteristic laminar and columnar organization of M1 and the barrel field of S1).

Within the previously defined ROIs, the immunoreactive area for GAD67 and PV was measured on thresholded images of each marker channel. The resulting values were expressed as the area fraction (%) of marker-positive pixels relative to the total ROI area, providing a quantitative index of $GAD67^+$ and PV^+ signal density within each cortical layer. This approach enabled the assessment of GABAergic enzyme and interneuronal marker distribution across the sensorimotor cortex, thereby contextualizing the cortical area–specific alterations shown in **Figure 3e–g**. At least

four coronal sections per animal were analysed, with four animals included per group. Counts were averaged across fields and sections to obtain a single mean value per animal. The same analysis were performed for quantification of GAD65 immunopositive profile density in SM CRTX (**Supplementary Figure 3**). At least four coronal sections per animal were analysed, with three animals included per group. Counts were averaged across fields and sections to obtain a single mean value per animal.

Subsequently, in the same coronal brain sections, quantitative analysis of GAD67+ and PV+ cell number was achieved by manually counting GAD67+/PV+ neuronal somas in confocal photomicrographs. In details, $GAD67^+$ and PV^+ neurons were manually counted in confocal images from defined cortical regions using consistent imaging and analysis parameters. Cell counting at P12 included six WT and six SMA mice. For each mouse, at least four coronal sections were analysed, and at least four confocal fields per section were counted in the M1 and S1 cortices. Counts were averaged across fields and sections to obtain a single mean value per animal. Further morphological analysis of PV+ INs was performed in these latter confocal images by semi-automated segmentation of PV+ cells. In detail, in thresholded images, the binary soma mask was obtained with the *Analyze Particle* command, determining the soma size range for PV+ object staining and then measuring each soma size (area) value. Next, the neurite skeleton was created (after soma subtraction) with the *Skeletonize* command and evaluated for quantitative IN shape analysis (using the *Analyze Skeleton* tool). Measurements of mean values for soma area, branch length, and number of endpoints are reported. Cell counting at P12 included six WT and six SMA mice. For each mouse, at least four coronal sections were analysed, and at least four confocal fields per section were counted in the M1 and S1 cortices. Counts were averaged across fields and sections to obtain a single mean value per animal. Quantitative analysis of PV^+ cell number across SM cortex layers was performed on digital slide scanner microscope images, following the same workflow described above for the quantification

of GABA$^+$ neurons. The interneuron (IN) number was obtained by normalizing the number of PV$^+$ neuronal somata (manually counted) to the number of DAPI$^+$ nuclei (calculated using the Watershed and Analyze Particles tools) in thresholded images. Analyses were conducted across different cortical layers within the selected ROIs of M1 and S1, defined according to the atlas-derived overlay masks and confirmed by DAPI staining. Cell counting at P12 included five WT and five SMA mice. For each mouse, at least four coronal sections were analyzed, and at least four confocal fields per section were counted in the M1 and S1 cortices. Counts were averaged across fields and sections to obtain a single mean value per animal. For GABAergic synapse analysis in motor CRTX layers (**Fig. 4E-H**), the image segmentation and quantification of inhibitory synapse puncta was performed readapting protocol from [9,10]. Z-stack images were analysed in the maximum projections of selected optical planes in confocal acquisitions (**Fig. 4E**) or in single optical planes in optical scanning microscope acquisitions (**Fig.4 H**) including the entire cell body of the MAP2+ neuron and all synapse boutons (GAD+ and Gephyrin+). Briefly, the images, processed in all channels for noise reduction and smoothing, were scanned firstly by selecting optical planes and then, for each channel, the ROI of the single neuron to be analysed was isolated in separate image, then a colour threshold was automatically set to identify: i) the cell body (MAP2+); and ii) the synaptic boutons (GAD+ and/or Gephyrin+). For the presynaptic GAD+ and postsynaptic Gephyrin+ boutons, the colour thresholding allowed boutons segmentation into background cleaned and detectable puncta (cluster of synaptic proteins GAD and/or Gephyrin labelled), moreover a watershed-based method was used to further separate puncta on the local minima of the pixel grey values. Following, masked binary images for cell body and puncta were created and merged, then an ROI was selected for the perimeter of the cell body, and GAD+ or Gephyrin+ synapse puncta contacting the soma were counted with the “Analyze Particles” tool (ensuring the minimum size for presynaptic structures as 0.20 μm and for postsynaptic ones as size 0.3 μm). The quantification of inhibitory synapse complexes (**Fig. 4H**) used a colocalization threshold based on the combined radius of each spot, identifying pre- and post-synapses only when juxtaposed [9]. The same criteria for each channel processing were applied to all images (neurons) from

the same experiment, at least 60 neurons were analysed from three different animals per group in single layers, Image acquisition for immunofluorescence analysis primary neuron and astrocytes was performed at *DIV15* with a Leica TCS SP5 laser scanning confocal microscope (Leica Microsystems), with Zeiss LSM 980 confocal microscope (Carl Zeiss AG), with Zeiss Apotome 3 optical scanning microscope (Carl Zeiss AG) and with the Incucyte® Live-Cell Analysis System (Sartorius). Confocal acquisitions were performed on optical sections using a 40× oil-immersion objective (N.A. 1.30), across a 6-μm section thickness, with z-steps of 0.5 μm, a resolution of 1024 × 1024 pixels, and a scanning frequency of 200 Hz. Exceptions were made for the analysis of inhibitory synapses in primary cortical neurons **(Fig. S5)**, for which optical sections were acquired using either a 40× (N.A. 1.30) or a 63× oil-immersion objective (N.A. 1.40), across a 5-μm section thickness, with z-steps of 0.5 μm, a resolution of 2048 × 2048 pixels, pixel time: 5; frame time: 40.27 sec. Optical scanning microscope acquisition were performed in high-resolution optical sectioning on sections scanned using a 20x objective (N.A. 0.8) objective, throughout 6 μm section thickness at 0.5 μm z-steps, with a resolution of 1024 × 1024 pixels. Widefield images were obtained in Incucyte microplate reader with a 20x objective (N.A. 0.8) objective. GABA and/or SMN mean signal intensity in primary neuron and astrocytes were performed on maximum projections Z-stacks images and analysis. GABA/SMN mean signal intensity values were obtained by manually selecting, in thresholded images, the cellular perimeter of neurons and astrocytes (according to MAP2 and GFAP staining, respectively) as regions of interest (ROI), measuring there the mean pixel intensity.Analyses included at least 100 cells from six independent cultures for each group. GABA signal was further assessed using two complementary approaches (**Fig. S1 c**): (i) percentage of GABA-positive pixel area relative to the total analysed field, and (ii) percentage of GABA-positive pixel area within selected ROI of MAP2$^+$ neurons. In addition, the proportion of GABA$^+$ neurons in WT and SMA co-cultures was quantified by semi-automated counting of the cellular perimeter of MAP2$^+$ neurons in thresholded images and expressed as a percentage of the total MAP2$^+$ population; it was approximately 25%, consistent with expected ranges for cortical primary cultures11,12 (Fig. S1 c). Analysis of inhibitory

synapses in primary cortical neurons was performed using the same protocol applied for GABAergic synapse analysis in motor cortex layers, readapted from [9,10]. Analyses included at least 100 cells from three independent cultures for each group.

Immunoblotting analysis

For the analysis of the protein expressions, SM CRTX samples from P5 and P12, the tissue lysis, protein quantification and denaturation and the following SDS-PAGE and western blot (WB) assays, were performed according to [13]. Briefly, SM CRTX samples were homogenized on ice in radioimmunoprecipitation assay buffer (RIPA lysis buffer) (Merck Life Sciences) supplemented with 1 mM PMSF, 1 mM DTT, 2 mM sodium orthovanadate (ThermoFisher Scientific) and 1 × complete™ Protease Inhibitor Cocktail (Merck Life Sciences). After incubation on ice (20 min), the sample homogenates were centrifuged (14,000 × g for 20 min at 4 °C) and the supernatant was collected (total protein solution). Total protein concentration was assayed on supernatants using Bradford reagent (Bio-Rad). Protein denaturation was performed with NuPAGE® LDS Sample Buffer supplemented with NuPAGE® Sample Reducing Agent (ThermoFisher Scientific) (95 °C, 5 min). SDS–PAGE and transfer were performed on 4–20% MiniPROTEAN® TGX™ Precast Protein Gels and Trans-Blot® Turbo™ mini nitrocellulose membranes using a TransBlot® Turbo™ transfer System (Bio-Rad), respectively. Nonspecific binding sites were blocked using 5% non-fat dried milk in PBS-0.2% Tween-20 (Merck Life Sciences) (PBS-T) (1 h, room temperature (RT), under shaking). All membranes were incubated overnight at 4 °C under shaking with primary antibody solutions (diluted in 2% nonfat dried milk in PBS-T), and the day after with HRP-conjugated secondary antibody solutions (diluted in 2% nonfat dried milk in PBS-T) (1 h, RT under shaking). All the relative details and dilutions are listed in **Table S1**. Immunolabeling was detected with Clarity™ Western ECL Blotting Substrates (Bio-Rad) using the ChemiDoc™ imaging system (Bio-Rad). The densitometric quantitation of bands intensity for the protein levels was calculated with reference to Vinculin (VINC) protein levels or to Total Protein Content (Ponceau staining) used as loading

control, using the Fiji software (Image J, NIH); the full, uncropped gels and blots are provided in the supplementary file: "**Additional File 2: Full unedited immunoblotting membranes**."

Patch clamp

Coronal slices (300 µm-thick) of the primary motor cortex were cut on a vibratome in ice-cold, oxygenated cutting solution. Slices were then transferred in artificial cerebrospinal fluid (ACSF) at 34 °C for 1 h and then at room temperature until use. ACSF contained (in mM) 125 NaCl, 2.5 KCl, 25 $NaHCO_3$, 1 NaH2PO4, 25 Glucose, 1 $MgCl_2$, 2 $CaCl_2$. Recording pipettes (4–6 MΩ) were made from borosilicate glass capillaries and filled with intracellular solution containing (in mM) 140 CsCl, 10 HEPES, 2 MgCl2, 4 Na2ATP, 0.4 NaGTP (pH 7.2). The intracellular solution also contained 20 µM of Alexa Fluor 568 (Thermo Fisher Scientific, Waltham, MA) and 2 mg/ml of biocytin (Sigma) for post-recording evaluation of the neuronal morphology. Whole-cell current clamp recordings were obtained from visually identified pyramidal neurons (layer 5). Recordings were carried out using a MultiClamp 700B amplifier (Molecular Devices, Sunnyvale, CA) and acquired with a Digidata digitizer (Molecular Devices). Spontaneous inhibitory postsynaptic currents (sIPSCs) were recorded in voltage clamp configuration at a holding potential (Vh) -70 mV in presence of NBQX (10 µM; Sigma) to block AMPA/Kainate mediated transmission. Under these experimental conditions E_{Cl} is near 0 mV and IPSCs are inwardly directed. Miniature IPSCs were subsequently isolated by blocking action potential-dependent transmission with 1 µM tetrodotoxin (TTX; Tocris Cookson, Bristol, UK). IPSCs were completely blocked by 25 µM of bicuculline, demonstrating the GABAergic nature of the currents. Series resistance (Rs) was monitored throughout the recordings. Recordings were discarded if Rs changed by more than 20% during the experimental procedure. sIPSCs/ miniature inhibitory postsynaptic currents (mIPSCs) were analysed off-line by Mini Analysis software (Synaptosoft Inc., Decatur, GA). sIPSCs/mIPSCs frequency and amplitude were sampled for periods of 100 s.

Bioinformatic analysis

For the bioinformatic analysis we reinterrogated a published dataset GEO: GSE102204 from the work of Bernabò et al., (2017) performed on tissue samples harvested from “Taiwanese” SMA mice at two different symptomatic disease stages [14]. Herein the authors explored the global consequences of SMN deficiency on gene expression, at the transcriptional as well as the post transcriptional levels by RNA sequencing not only total mRNA expression but also polysomal mRNA engagement. We selected only samples tagged as polysomal and late, including 3 cases and 3 controls. Genes were filtered for RPKM>1in at least 3 samples, which left us with 14464 genes to be tested for differential polysomal expression between SMA and control samples. RPKM were transformed as log2(RPKM+1) and a linear model was used to estimate coefficients, interpreted as log2-fold changes. Then we adopted the R package clusterProfiler (v 4.12.6) to run a GSEA analysis on two different lists of genes. The first consisted of all the genes annotated to the Gene Ontology category ‘GABAergic neuron differentiation’ (GO:0097154). The second was obtained from the Linnarsson Atlas of the Adolescent Mouse Brain (Mousebrain.org) (PMID:30096314), by merging the genes highly enriched in cortical or hippocampal inhibitory neuron clusters (TEINH4-TEINH12).

RNAi SMN expression in primary cortical neurons

Isolation and co-culture of primary WT mouse neurons and astrocytes were performed as previously described, except for slight modifications allowing applications of the SMN RNAi protocol. From days *in vitro* (*DIV) 7*, half-media changes were performed every 3/4 days with free P/S culture media, then, at *DIV 14,* cells media were completely replaced by adding scramble (SCR) or siRNA solutions to cells. In detail, WT neuron cultures were transfected with LipofectAMINE 2000 (Invitrogen) according to the manufacturer's protocols and optimized amounts of SCR and SMN siRNA (66µM) (Horizon), for a transfection duration (24 hours) set to minimize toxicity and maximize transfection efficiency. At the end of the treatment (*DIV15*), cells were fixed to allow

following immunostaining analyses (a schematic representation of the experimental workflow is provided in **Figure S1 a**). Further assessment of treatment-related toxicity was performed by quantifying the number of $GABA^+$ neurons in high-magnification fields selected for experimental analysis. The proportion of $GABA^+$ neurons was calculated relative to the total number of $MAP2^+$ neurons in each field and compared between untreated (NT), scrambled siRNA–treated (SCR), and siSMN-treated co-cultures. This analysis revealed no significant differences among groups (approximately 40% of total neurons), confirming that the applied treatments did not induce general cytotoxicity or selective loss of GABAergic neurons (**Supplementary Figure 1d**).

Nusinersen treatment of primary cortical cells

To investigate chronic effects on neuronal function, SMA mice primary cortical neurons and astrocytes co-cultures were treated with 200 nM nusinersen (Nusinersen sodium, MedChem Express, Cat. No.: HY-112980A) dissolved in water, added directly to the culture medium every three days from day *in vitro* (*DIV*) 7 to *DIV15*, with half of the medium replaced at each administration. This concentration was chosen as it represents the maximum dose previously tolerated by SMA patient-derived fibroblasts [15] (a schematic representation of the experimental workflow is provided in **Fig. S1 a**). Previous experiments showed that primary cortical neurons tolerated transfection for up to 24 h (Fig. 6a–c). Therefore, passive delivery without transfection was used for chronic treatment, allowing drug administration from *DIV7* to *DIV15*, encompassing the period of neuronal structural and synaptic functional maturation *in vitro*[16], to assess effects on neurotransmission. Although only a fraction of the oligonucleotide enters the cells, previous studies have shown that passive uptake of antisense oligonucleotides is sufficient to achieve functional modulation of target genes in primary cortical neurons [17]. Control SMA neurons received an equivalent volume of water as vehicle and were maintained under identical treatment conditions, including medium replacement schedule, timing, and handling. At the end of the treatment, cells were lysed for protein extraction or fixed to allow following immunoblotting and immunostaining analyses, respectively.

Declaration of LLM tools technologies in the writing process

During the creation of this work, the authors occasionally used ChatGPT-4.0 to refine the language and enhance readability. The content was then thoroughly reviewed and revised by the authors, who take full responsibility for the final version.

Table S1. List of primary and secondary antibodies used in immunofluorescence and immunoblotting experiments.

Antibodies	IF/WB	Target	Reference	Host	Provider	Dilution
Primary	IF	GABA	A2052	Rabbit	Merck	IHC: 1:500<br>ICC: 1:1000
	IF	MAP2	AMab5392	Chicken	Abcam	IHC, ICC: 1:2000
	IF	GFAP	AB190288	Mouse	Abcam	IHC, ICC: 1:1000
	IF	Parvalbumin	PV27	Rabbit	Swant	IHC: 1:2000
	IF	Gephyrin	147011	Mouse	Synaptic System	IHC: 1:500
	IF	GAD65+GAD67	ab183999	Rabbit	Abcam	IHC: 1:500
	IF	SMN	610646	Mouse	BD Biosciences	ICC: 1:500
Fluorescent conjugated	IF	ExtrAvidin−Cy3	E4142	---	Sigma	IHC: 1:1000
	IF/WB	GAD67	MAB5406	Mouse	Merck	IHC: 1:500<br>WB: 1:1000

	IF/WB	GAD65	GAD 6; AB_528264	Mouse	DSHB	IHC: 1:500 WB: 1:1000
	WB	Parvalbumin [3C9]	ab277625	Mouse	Abcam	1:1000
	WB	GAT1	#37342	Rabbit	Cell Signaling	1:1000
	WB	GAT3 [EPR25153-38]	ab300559	Rabbit	Abcam	1:1000
	WB	SNAT5 (G-7)	sc-515813	Mouse	Santa Cruz Biotechnology	1:500
	WB	VGAT	131 011	Rabbit	Synaptic System	1:2000
	WB	Vinculin, clone HVIN-1	V9131-100UL	Mouse	Merck	1:2000
Secondary	IF	Cy™2 AffiniPure Donkey Anti-Mouse IgG (H +L)	AB_2340826		Jackson ImmunoResearch	1:400
	IF	Cy™2 AffiniPure Donkey Anti-Rabbit IgG (H + L)	AB_2340612		Jackson ImmunoResearch	1:400
	IF	Cy™3 AffiniPure Donkey Anti-Mouse IgG (H + L)	AB_2340813		Jackson ImmunoResearch	1:400
	IF	Cy™3 AffiniPure Donkey Anti-Rabbit IgG (H + L)	AB_2307443		Jackson ImmunoResearch	1:400

	IF	Alexa Fluor® 647 AffiniPure™ Donkey Anti-Chicken IgY (IgG) (H+L)	703-605-155	Jackson ImmunoResearch	1:400
	IF	DyLight™ 405 AffiniPure™ Donkey Anti-Chicken IgY (IgG) (H+L)	703-475-155	Jackson ImmunoResearch	1:400
	WB	GOAT anti-mouse IGG HRP	1706516	Biorad	1:10000
	WB	GOAT anti-rabbit IGG HRP	1706515	Biorad	1:10000

Bibliography for supplementary Materials and Methods

1. Le, T. T. *et al.* SMNDelta7, the major product of the centromeric survival motor neuron (SMN2) gene, extends survival in mice with spinal muscular atrophy and associates with full-length SMN. *Hum. Mol. Genet.* **14**, 845–857 (2005).

2. Cottam, N. C., Harrington, M. A., Schork, P. M. & Sun, J. No significant sex differences in incidence or phenotype for the SMNΔ7 mouse model of spinal muscular atrophy. *Neuromuscul. Disord.* **37**, 13–22 (2024).

3. Meeker, N. D., Hutchinson, S. A., Ho, L. & Trede, N. S. Benchmarks Method for isolation of PCR-ready genomic DNA from zebrafish tissues. **43**, 4–6 (2007).

4. Valsecchi, V., Boido, M., De Amicis, E., Piras, A. & Vercelli, A. Expression of muscle-specific MiRNA 206 in the progression of disease in a murine SMA model. *PLoS One* **10**, 1–

17 (2015).

5. Goshi, N., Morgan, R. K., Lein, P. J. & Seker, E. A primary neural cell culture model to study neuron , astrocyte , and microglia interactions in neuroinflammation. 1–16 (2020).

6. Chapman, C. A. R. *et al.* Nanoporous gold as a neural interface coating: effects of topography, surface chemistry, and feature size. *ACS Appl. Mater. Interfaces* **7**, 7093–7100 (2015).

7. D'Errico, P. *et al.* Selective vulnerability of spinal and cortical motor neuron subpopulations in delta7 SMA mice. *PLoS One* (2013) doi:10.1371/journal.pone.0082654.

8. Schneider, C. A., Rasband, W. S. & Eliceiri, K. W. NIH Image to ImageJ: 25 years of image analysis. *Nat. Methods* **9**, 671–675 (2012).

9. Favuzzi, E., Huang, S., Saldi, G. A., Datta, S. R. & Stevens, B. GABA-receptive microglia selectively sculpt developing inhibitory circuits ll ll Article GABA-receptive microglia selectively sculpt developing inhibitory circuits. 4048–4063 (2021) doi:10.1016/j.cell.2021.06.018.

10. Zhou, J. *et al.* The neuronal pentraxin Nptx2 regulates complement activity and restrains microglia-mediated synapse loss in neurodegeneration. *Sci. Transl. Med.* **15**, eadf0141 (2023).

11. Sahara, S., Yanagawa, Y., O'Leary, D. D. M. & Stevens, C. F. The fraction of cortical GABAergic neurons is constant from near the start of cortical neurogenesis to adulthood. *J. Neurosci. Off. J. Soc. Neurosci.* **32**, 4755–4761 (2012).

12. Dzyubenko, E. *et al.* Inhibitory control in neuronal networks relies on the extracellular matrix integrity. *Cell. Mol. Life Sci.* **78**, 5647–5663 (2021).

13. Januel, C. *et al.* Moxifloxacin rescues SMA phenotypes in patient - derived cells and animal model. *Cell. Mol. Life Sci.* (2022) doi:10.1007/s00018-022-04450-8.

14. Bernabò, P. *et al.* In Vivo Translatome Profiling in Spinal Muscular Atrophy Reveals a Role

for SMN Protein in Ribosome Biology. *Cell Rep.* **21**, 953–965 (2017).

15. Wijaya, Y. O. S. *et al.* High Concentration or Combined Treatment of Antisense Oligonucleotides for Spinal Muscular Atrophy Perturbed SMN2 Splicing in Patient Fibroblasts. *Genes (Basel).* **13**, (2022).

16. Petkova-Tuffy, A., Gödecke, N., Viotti, J., Korte, M. & Dresbach, T. Neuroligin-1 mediates presynaptic maturation through brain-derived neurotrophic factor signaling. *BMC Biol.* **19**, 215 (2021).

17. Tran, H. *et al.* Suppression of mutant C9orf72 expression by a potent mixed backbone antisense oligonucleotide. *Nat. Med.* **28**, 117–124 (2022).
