# Supplementary material for: Changes in the cortical GABAergic inhibitory system in a Spinal Muscular Atrophy mouse model
Source: Cell Death Dis. 2026 Feb 28;17(1):285. doi: 10.1038/s41419-026-08520-8 (PMC13031913; doi:10.1038/s41419-026-08520-8)
Supplement: Supplementary file 2 — Supplementary Figures 1 to 8 [file 41419_2026_8520_MOESM2_ESM.pdf]

Supplementary Figures 1 to 8 for: "Changes in the cortical GABAergic inhibitory system in a Spinal Muscular Atrophy mouse model"

Giovanna Menduti[1,2, #], Francesco Ferrini[3,4], Anna Caretto[1,2], Amber Hassan[5,6], Raffaella di Vito[6,7], Giada Beltrando[1,2], Davide Marnetto[2], Alessandro Usiello[6,7], Ferdinando Di Cunto[1,2], Marina Boido[1,2, *] and Alessandro Vercelli[1,2, *]

* These authors contributed equally to this work

# Corresponding author: Giovanna Menduti; full postal address: Neuroscience Institute Cavalieri Ottolenghi, Orbassano, 10043 Turin, Italy; telephone: + 39 011 670 6613; email: giovanna.menduti@unito.it

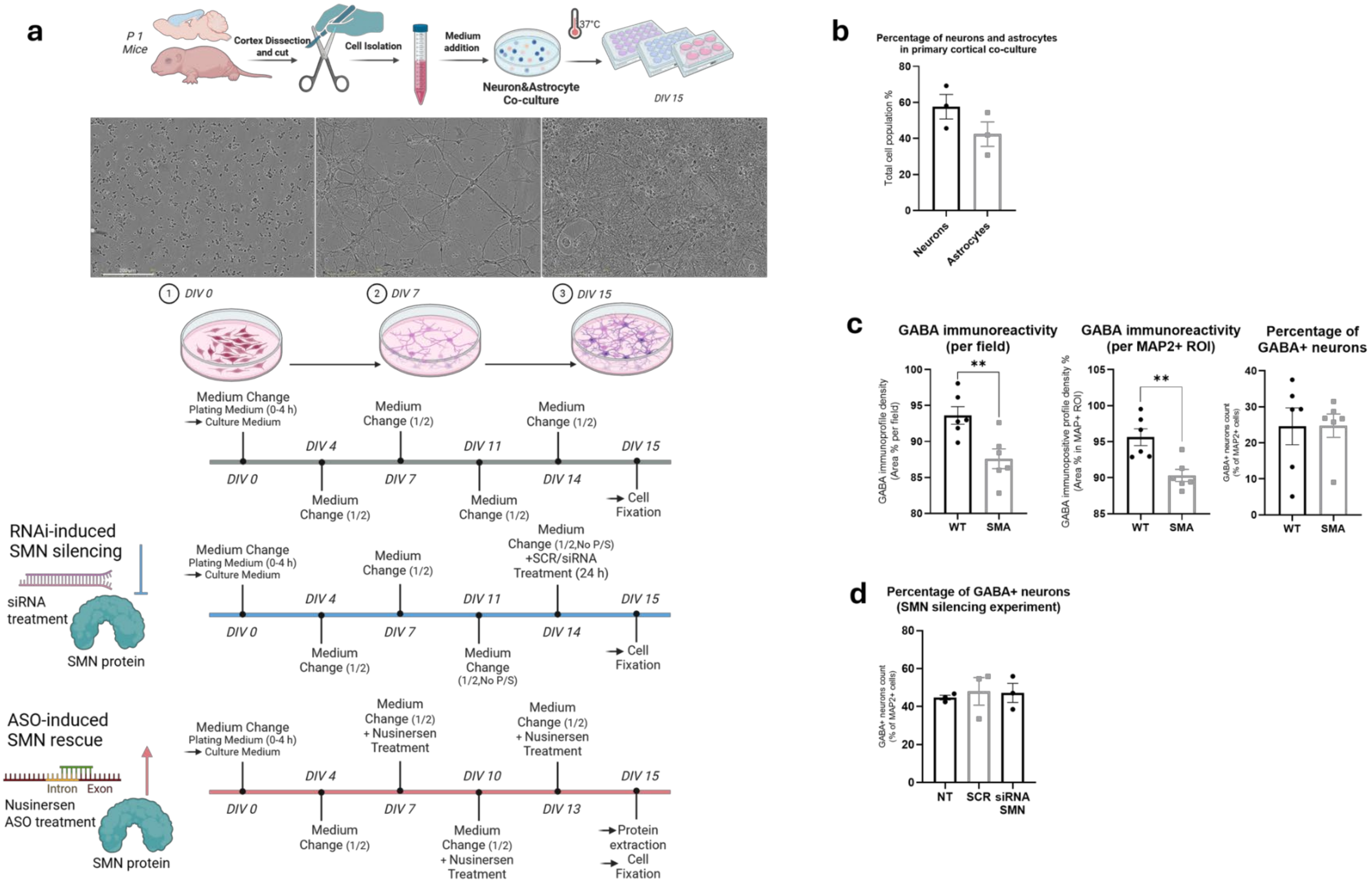

Supplementary Figure 1. Characterization of primary cortical neuron–astrocyte co-cultures and treatment workflows. a. Schematic overview of the experimental workflow for primary neuron–astrocyte co-cultures derived from WT and SMA mice. From top to bottom: schematic representation of cortical dissection from P1 mice, representative phase-contrast micrographs of cultured cells (scale bar: 200 µm), and a timeline illustrating medium changes and culture maintenance. The lower panels show the specific variations introduced for siRNA (SMN or scrambled) transfection and for antisense oligonucleotide (ASO) Nusinersen treatment. **b.** Quantification of cortical neurons and astrocytes in primary co-cultures. WT co-cultures were used as reference to assess the purity and reproducibility of the neuronal–astrocytic co-culture model. Data are presented as mean ± SEM, obtained from the analysis of at least 100 cells per culture from three independent WT co-cultures; each dot represents the mean percentage of neurons or astrocytes within an individual culture. **c.** Quantification of GABA immunoreactivity in WT and SMA cortical neuron–astrocyte co-cultures (complementary analysis to Fig. 1f). GABA signal was assessed using two complementary approaches (from left to right): (i) percentage of GABA-positive area relative to the total analysed field, and (ii) percentage of GABA-positive area within $MAP2^{+}$ neurons. The proportion of $GABA^{+}$ neurons within the $MAP2^{+}$ population was approximately 25% in both WT and SMA cultures. Data are presented as mean ± SEM; *Student's t*-test, $*p < 0.01$. Analyses included at least 100 cells from WT and SMA primary co-cultures (n = 6); each dot represents the mean value from an individual sample. **d.** Quantification of $GABA^{+}$ neurons in untreated, scrambled (SCR)–treated, and siRNA SMN-treated co-cultures (complementary analysis to Fig. 6a–c). Data are presented as mean ± SEM, obtained from the analysis of at least 100 cells per culture from three independent cultures for each group; each dot represents the mean value from an individual culture.

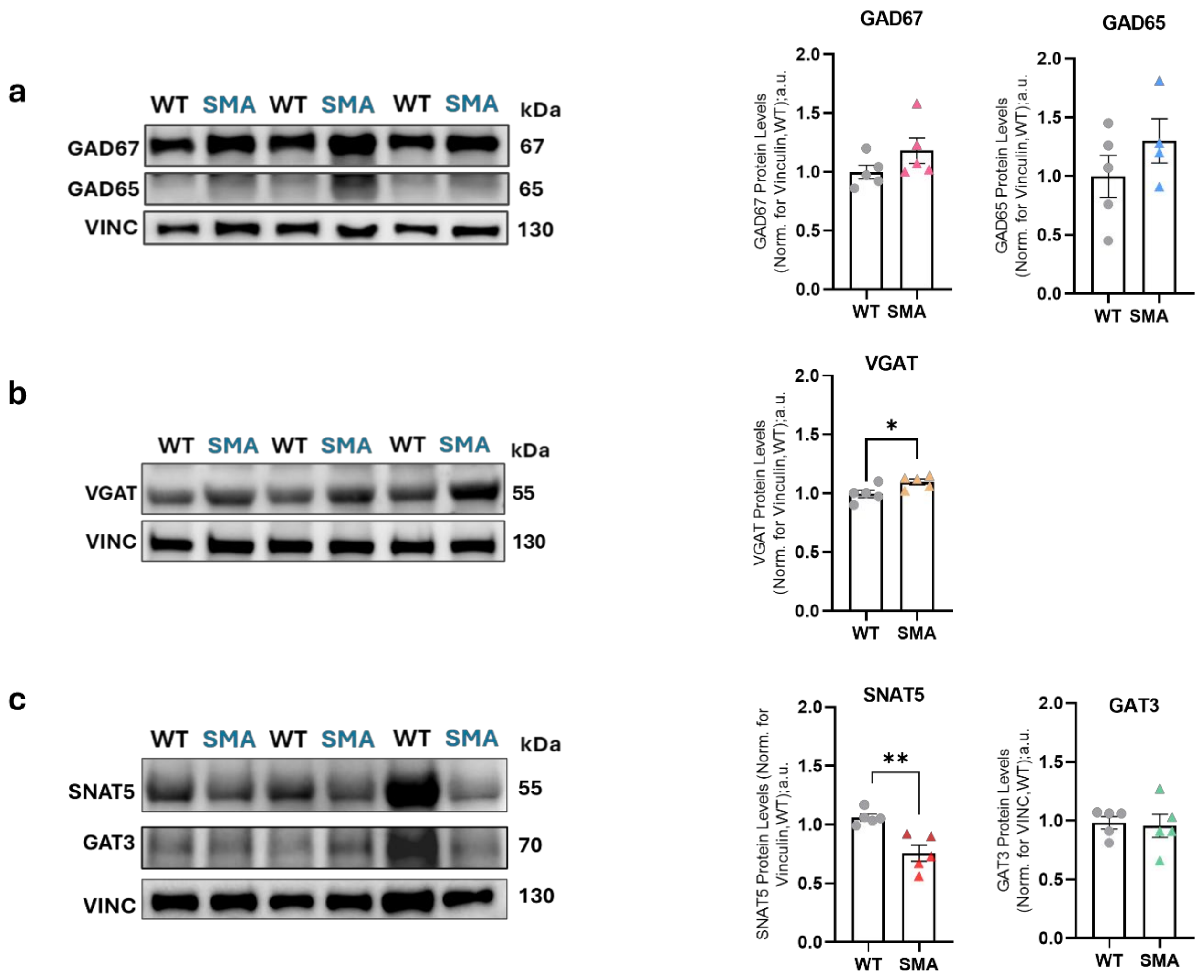

Supplementary Figure 2. GABAergic related proteins in P5 SM CRTX. Immunoblotting analysis of GABAergic molecular marker expressions in P5 SMA and WT SM CRTX. Representative densitometries and protein levels quantifications of GAD enzymes (**a**), of VGAT (**b**), of SNAT5 and GAT3 in SMAΔ7 SM CRTX homogenates compared to WT controls [Data are expressed as mean± SEM and normalized to VINC (referred as loading control) and to WT mean. Student's t-test, *$p < 0.05$, **$p < 0.01$; for GAD67, VGAT, SNAT5 and GAT3: WT and SMA mice: n=5; for GAD65: WT mice n=5 and SMA mice n=4].

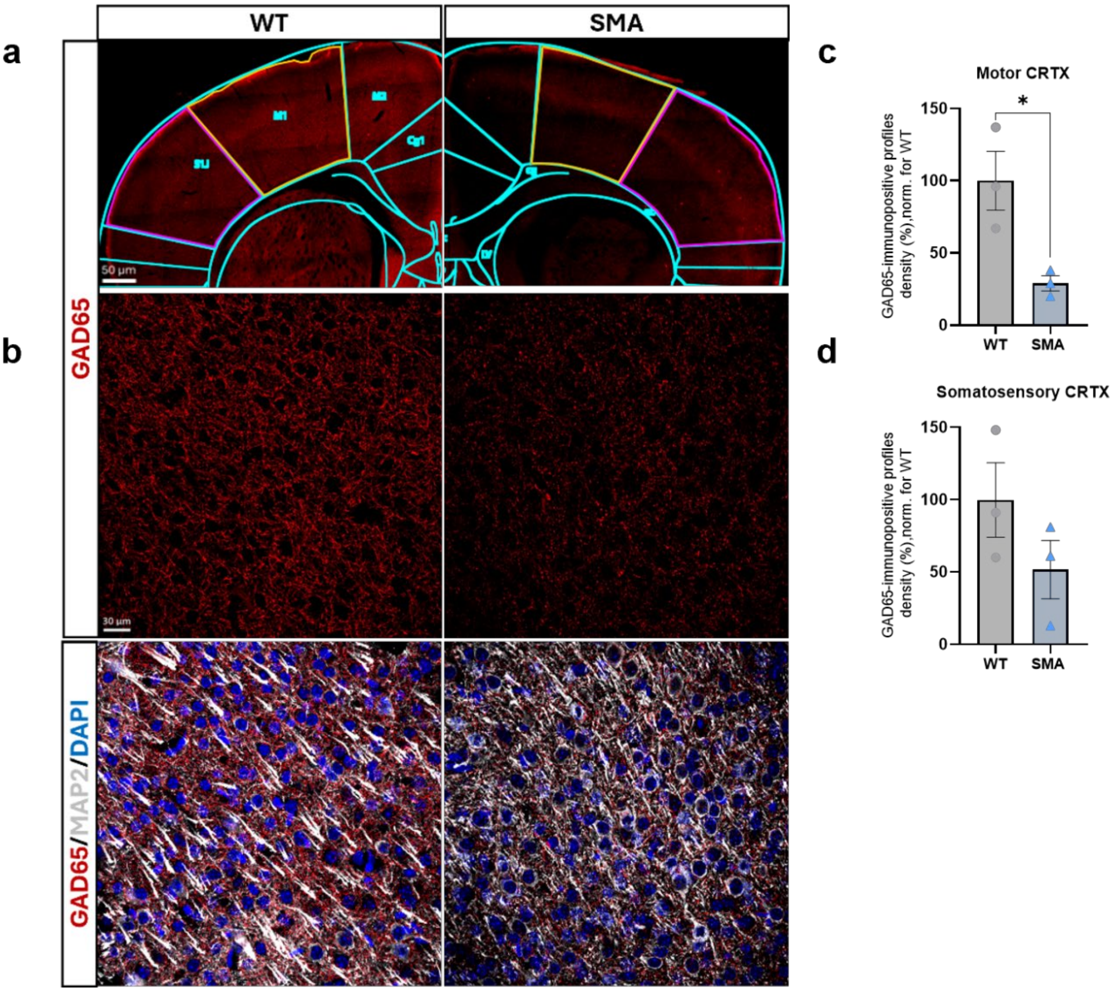

Supplementary Figure 3. GAD65 signaling is impaired in SMAΔ7 mice SM CRTX in late disease stage. a. Representative IF images of coronal brain sections from WT and SMA mice. Related brain atlas table are super-imposed (cyan) highlighting M1 (orange) and S1 (magenta) areas). Scale bar: 50 um. **b**. Representative confocal images of GAD65 (red) in WT and SMA mice SM CRTX. Neurons are labelled by MAP2 (grey) and nuclei are DAPI-stained (blue). Scale bar: 30 um. **c-d**. Analysis of GAD65 immunopositive profile densities (%) were performed in motor (**c**) and somatosensory (**d**) areas of SMA mice in comparison with WT ones (data are expressed as mean± SEM and normalized to WT mean, referred as control; Student's t-test, $^{*}p < 0.05$; WT and SMA mice: n=3).

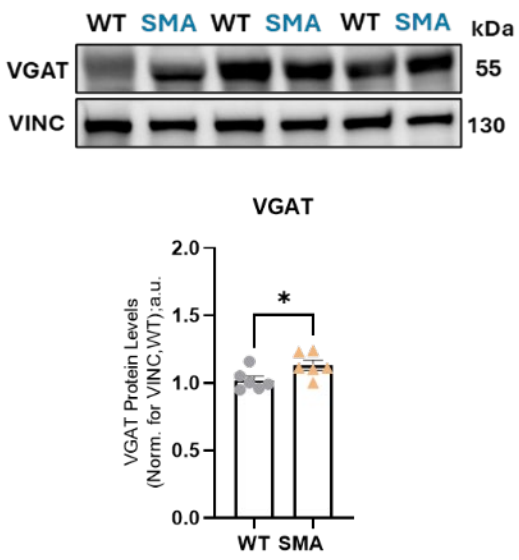

Supplementary Figure 4. VGAT expression is dysregulated in SMAΔ7 mice SM CRTX. Representative densitometry and protein level quantification of VGAT in SMA P12 SM CRTX samples compared to WT controls [Data are expressed as mean± SEM and normalized to VINC (referred as loading control) and to WT mean. Student's t-test, *p < 0.05; WT mice: n=6; SMA mice: n=6.

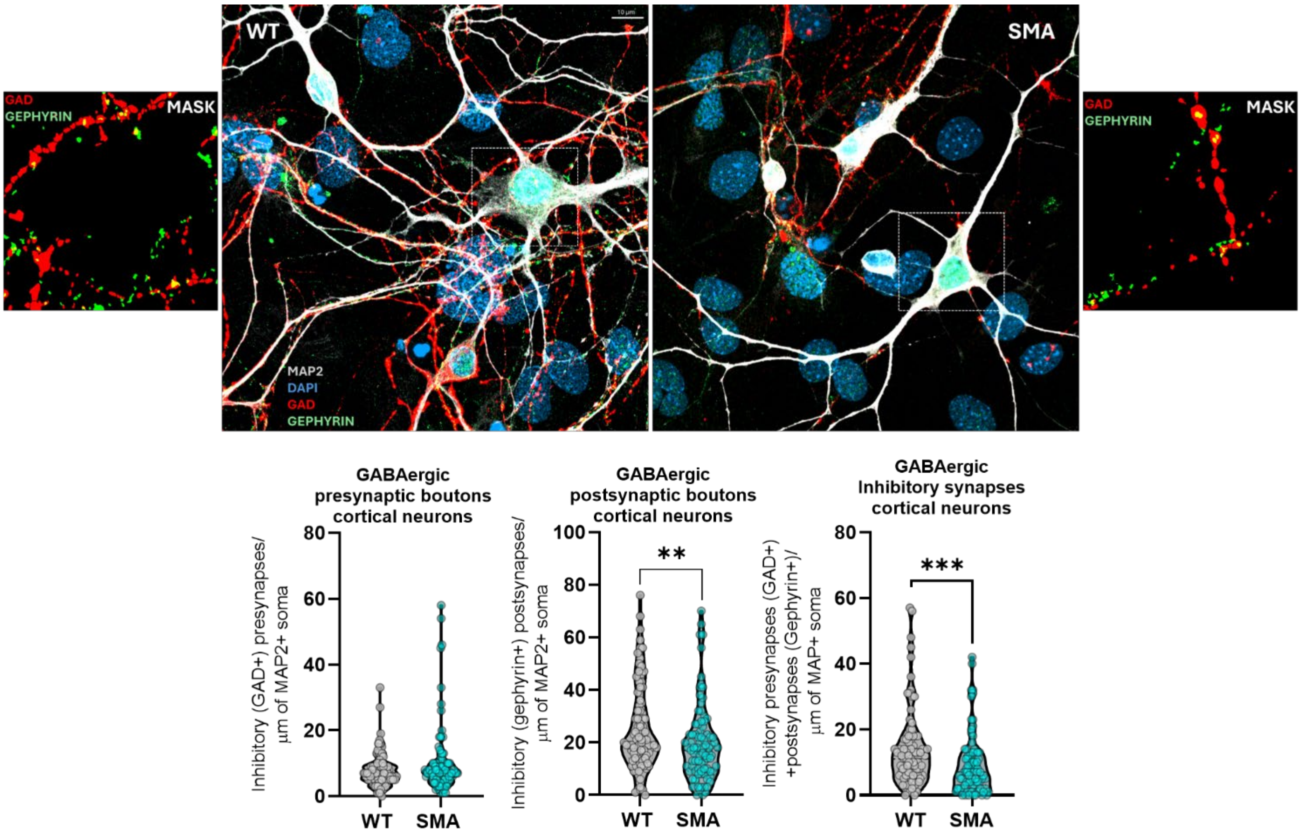

Supplementary Figure 5. Quantification of inhibitory synapses in primary cortical neurons. Confocal images of WT and SMA primary cortical neurons (scale bar: 10 µm) are shown, with binary masks for GABAergic pre-synapses (GAD$^+$, red) and post-synapses (gephyrin$^+$, green) displayed in the lateral insets. Inhibitory synaptic complexes (GAD$^+$/gephyrin$^+$ co-localization) were quantified based on their distribution along the MAP2$^+$(grey) neuronal soma perimeter. Violin plots (from left to right) show the cumulative distribution of GAD$^+$ and gephyrin$^+$ synapses contacting neurons, and of inhibitory synaptic complexes. Analyses included at least 100 cells from three independent cultures for each group. Each dot on the graph shows inhibitory synapse number from individual neuron. Statistical comparisons were performed using the Mann–Whitney test (**$p < 0.01$, ***$p < 0.005$).

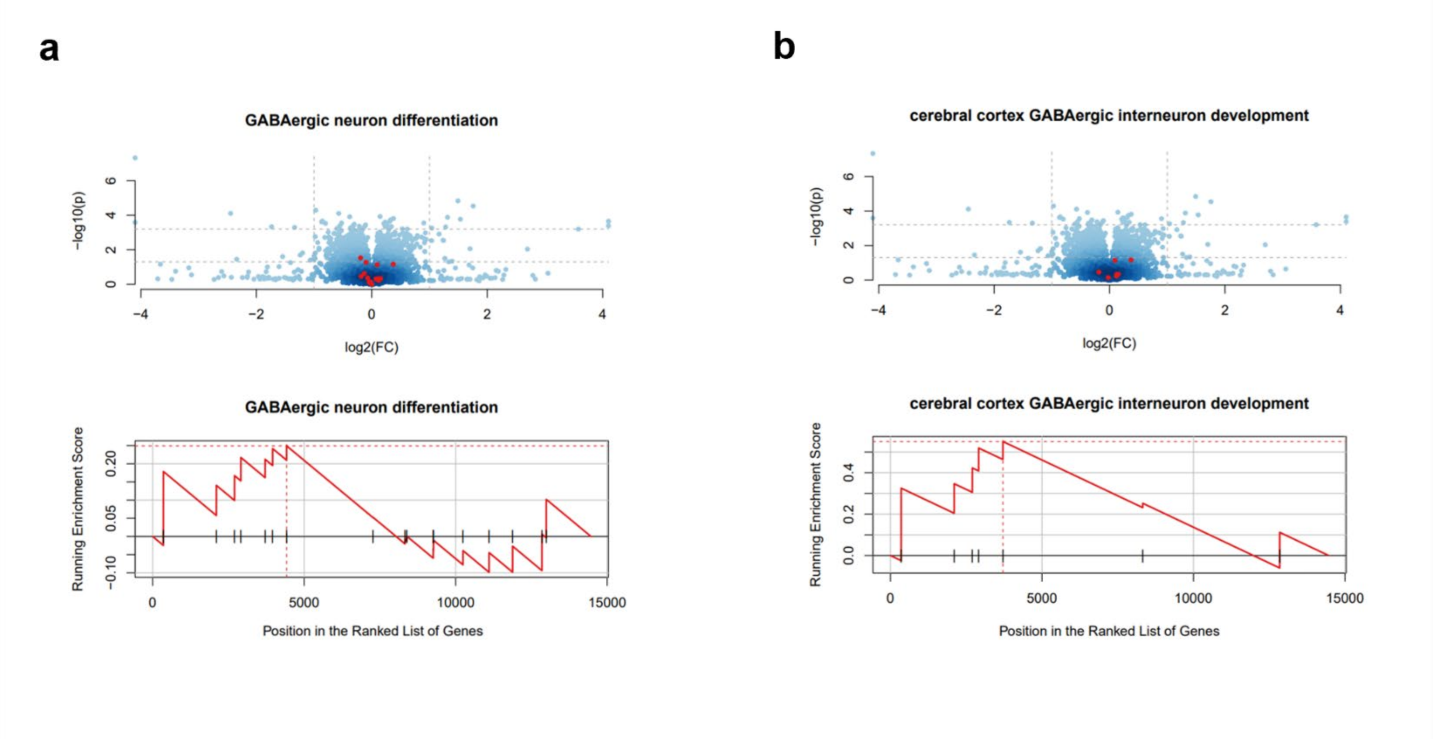

Supplementary Figure 6. GABAergic dysregulations in SMAΔ7 mice CRTX are not associated to neurodevelopmental issues. Volcano plot of differential polysomal mRNA abundance between SMA and healthy samples at late-symptomatic time point, from Bernabo' et al 2017. Each dot represents a gene, higher dot density is shown as darker color, while dashed lines mark |log2(FC)|=1 and significance, both at nominal (P=0.05) and at FDR=0.2 level, so that genes outside the marked area are traditionally considered differentially expressed at a lenient cutoff. Genes marked in red belong to the Gene Ontology category 'GABAergic neuron differentiation' (GO:0097154) (**a**)/ a hand-curated list of GABA-ergic interneuron markers (**b**). The plotting range has been reduced to |log2(FC)|=4 for visualization, with more extreme genes reported at the boundaries.

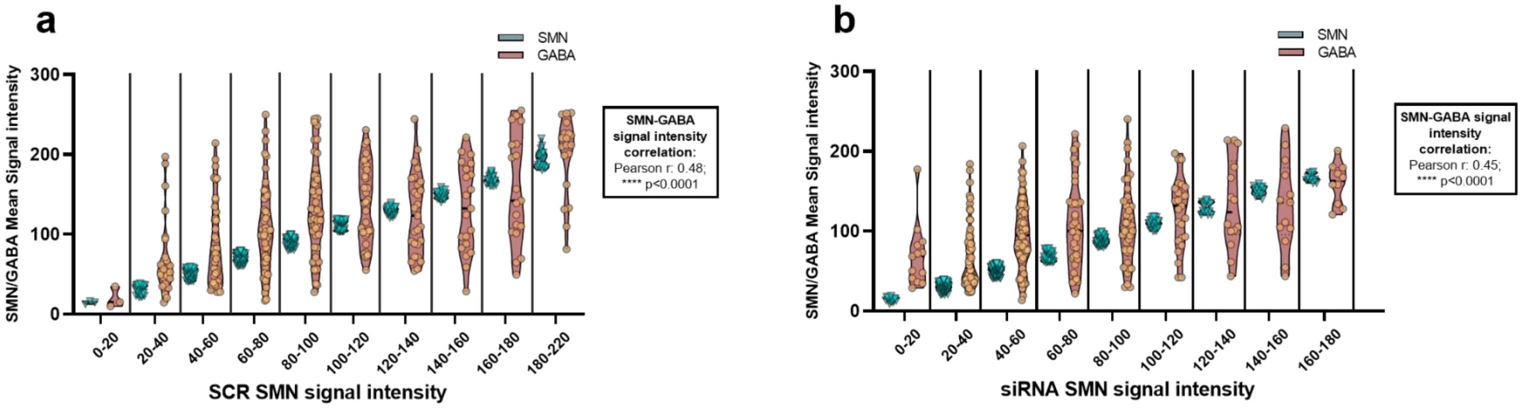

Supplementary Figure 7. SMN deficiency is correlated to dysregulations of GABAergic signaling in SMA neurons. Correlation analysis of neuronal variance of SMN and GABA signal intensity along increasing order gates of mean SMN signal intensity in SCR (**a**) and siRNA (**b)** neurons (individual dots) (Pearson r correlation test, ****p< 0.0001; n=3 mice primary neuron cultures per group).

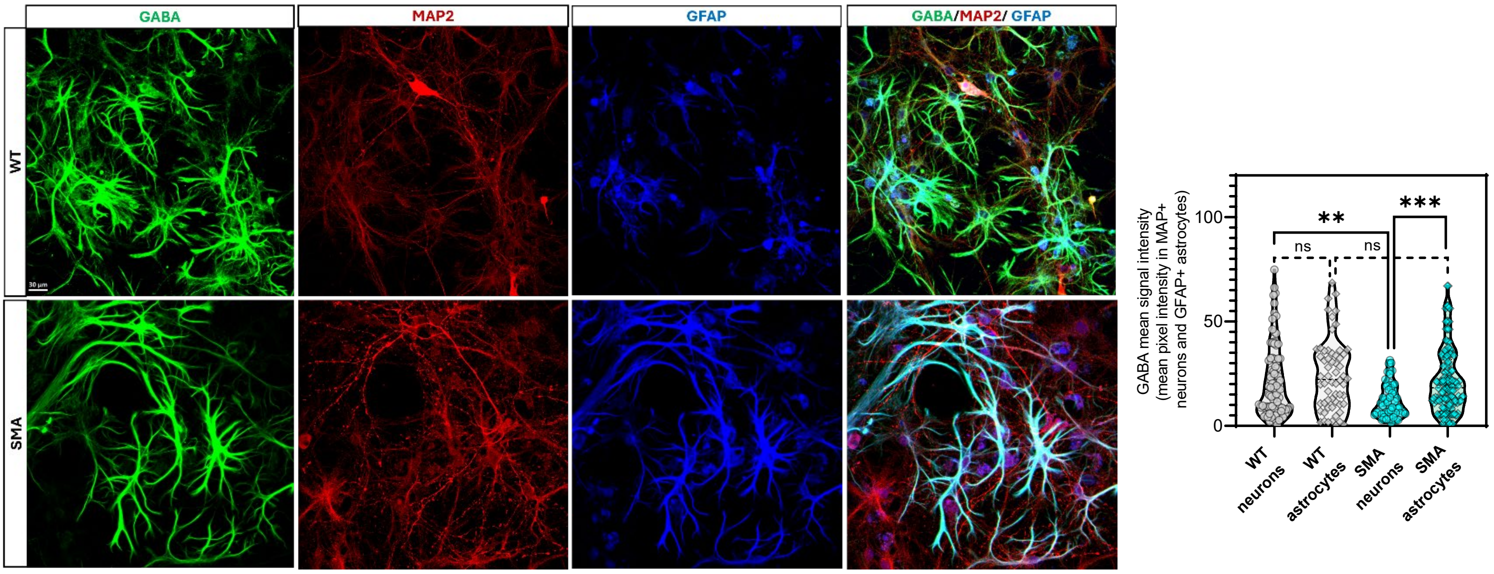

Supplementary Figure 8. GABAergic signaling in mice primary cortical cells is dysregulated in SMA neurons and astrocytes. Representative confocal immunofluorescence images (left) of WT and SMA primary cortical neurons (MAP2, red) and astrocytes (GFAP, blue) co-labelled for GABA (green). Scale bar: 30 µm. Quantification (right) of GABA mean intensity in neuron and astrocytes among WT and SMA cortical cells. Analyses included at least 100 cells from six independent cultures for each group. Each dot on the graph represents an individual cell (Mann-Whitney test, **p< 0.01; ***p< 0.005; WT and SMA mice: n=6 primary cell co-cultures).
