# Supplementary material for: Changes in the cortical GABAergic inhibitory system in a Spinal Muscular Atrophy mouse model
Source: Cell Death Dis. 2026 Feb 28;17(1):285. doi: 10.1038/s41419-026-08520-8 (PMC13031913; doi:10.1038/s41419-026-08520-8)

Full unedited immunoblotting membranes for: “Changes in the cortical GABAergic inhibitory system in a Spinal Muscular Atrophy mouse model”

Giovanna Menduti[1,2, #], Francesco Ferrini[3,4], Anna Caretto[1,2], Amber Hassan[5,6], Raffaella di Vito[6,7], Giada Beltrando[1,2], Davide Marnetto[2], Alessandro Usiello[6,7], Ferdinando Di Cunto[1,2], Marina Boido[1,2, *] and Alessandro Vercelli[1,2,*]

* These authors contributed equally to this work

# Corresponding author: Giovanna Menduti; full postal address: Neuroscience Institute Cavalieri Ottolenghi, Orbassano, 10043 Turin, Italy; telephone: + 39 011 670 6613; email: giovanna.menduti@unito.it

Legend:

SELECTED BANDS (WT/SMA)

PROTEIN MARKER

Protein Ladder

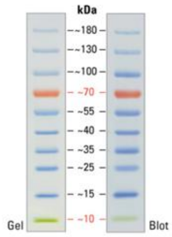

Pageruler prestained protein ladder 26616

Figure 3A

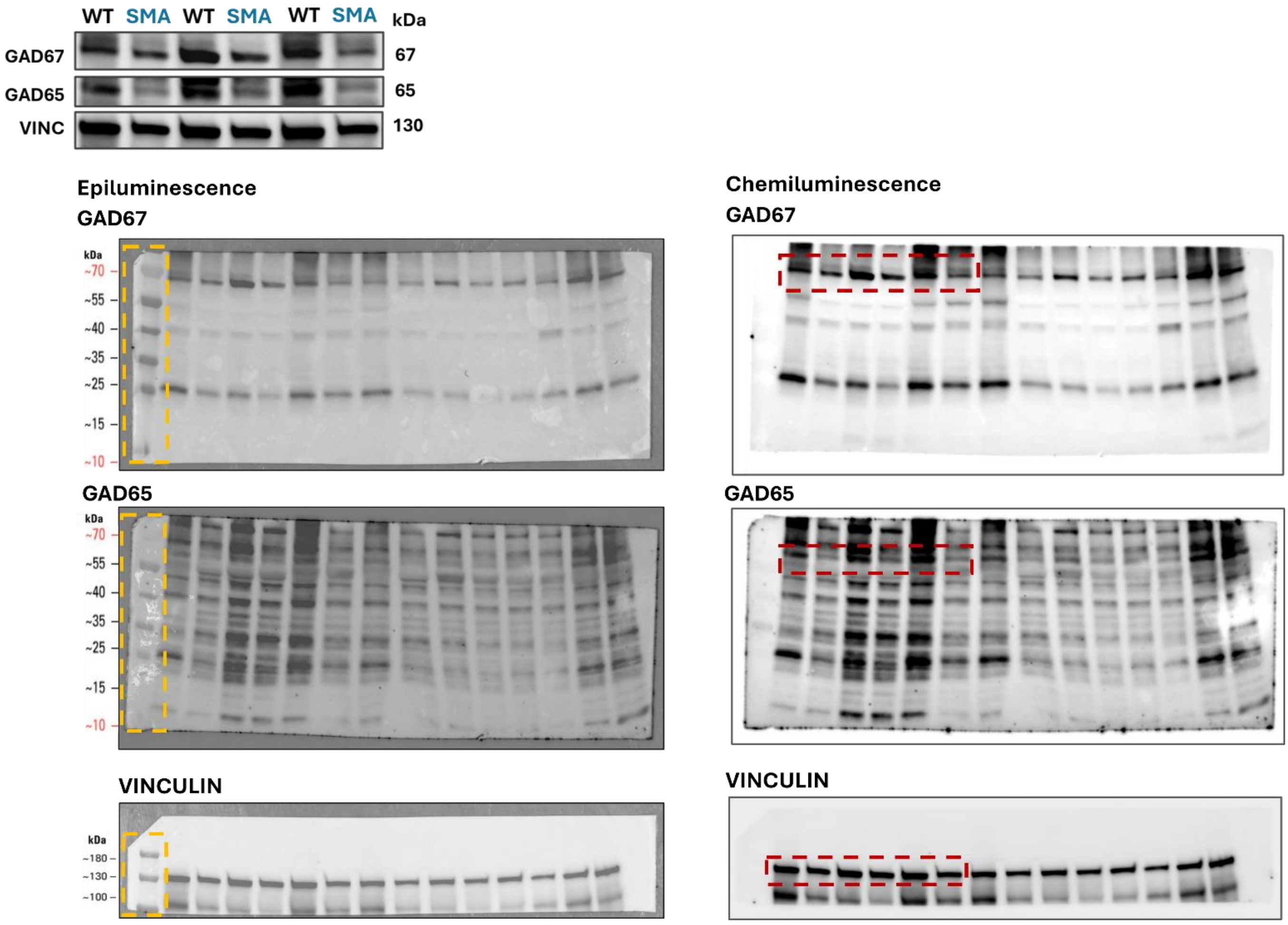

Figure 3B

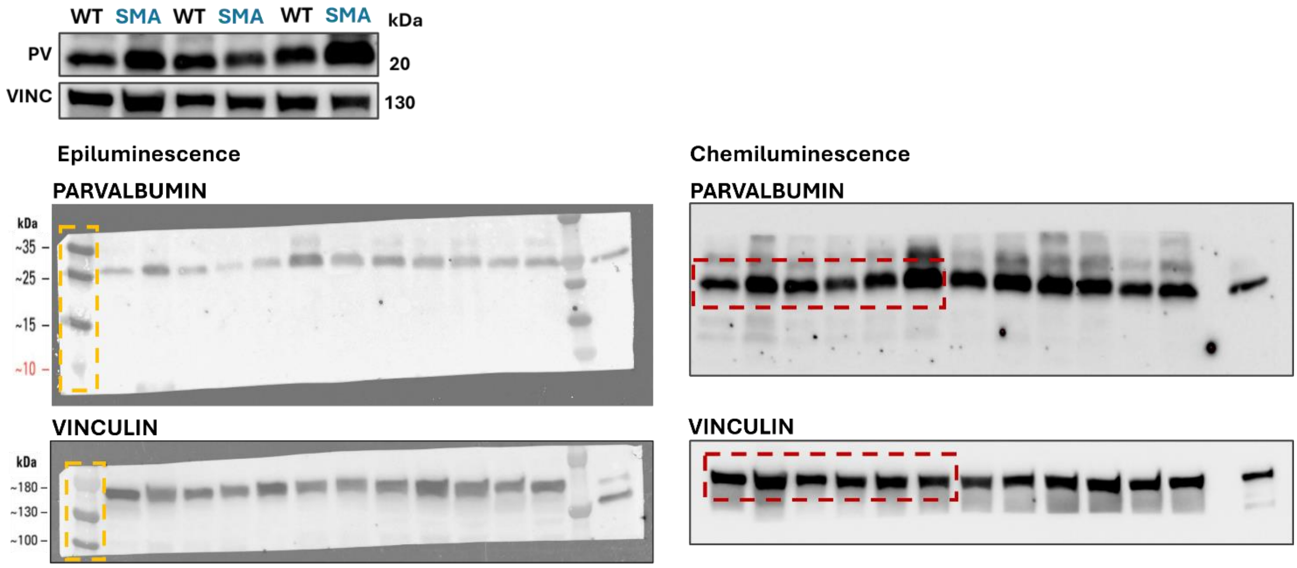

Figure 6D

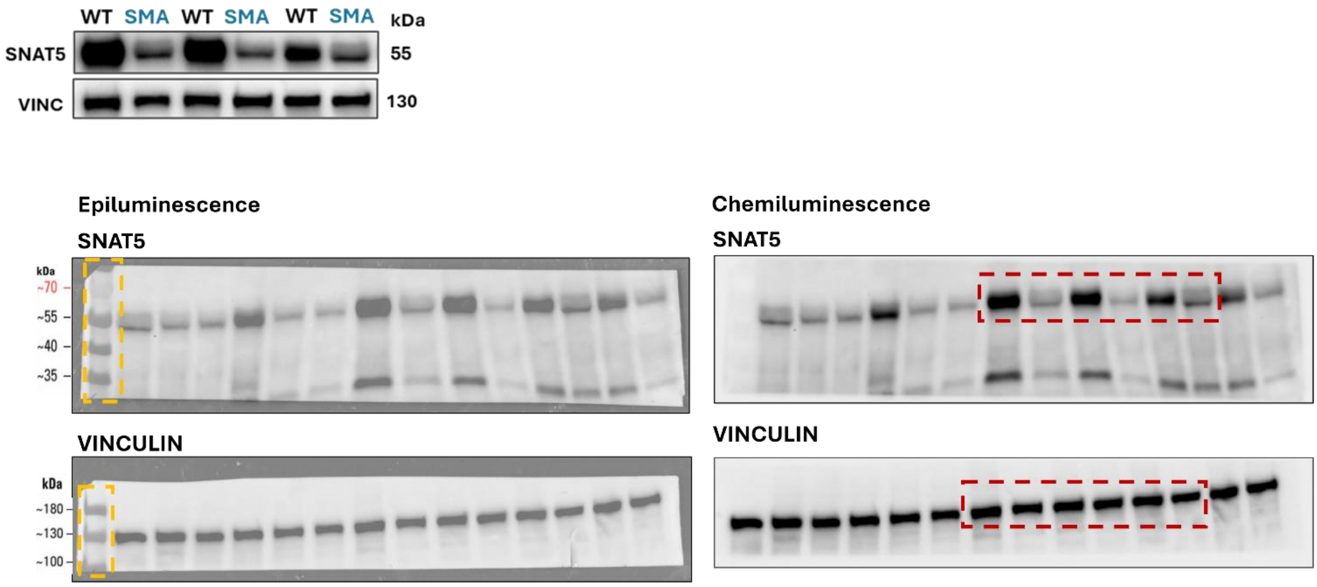

Figure 6E

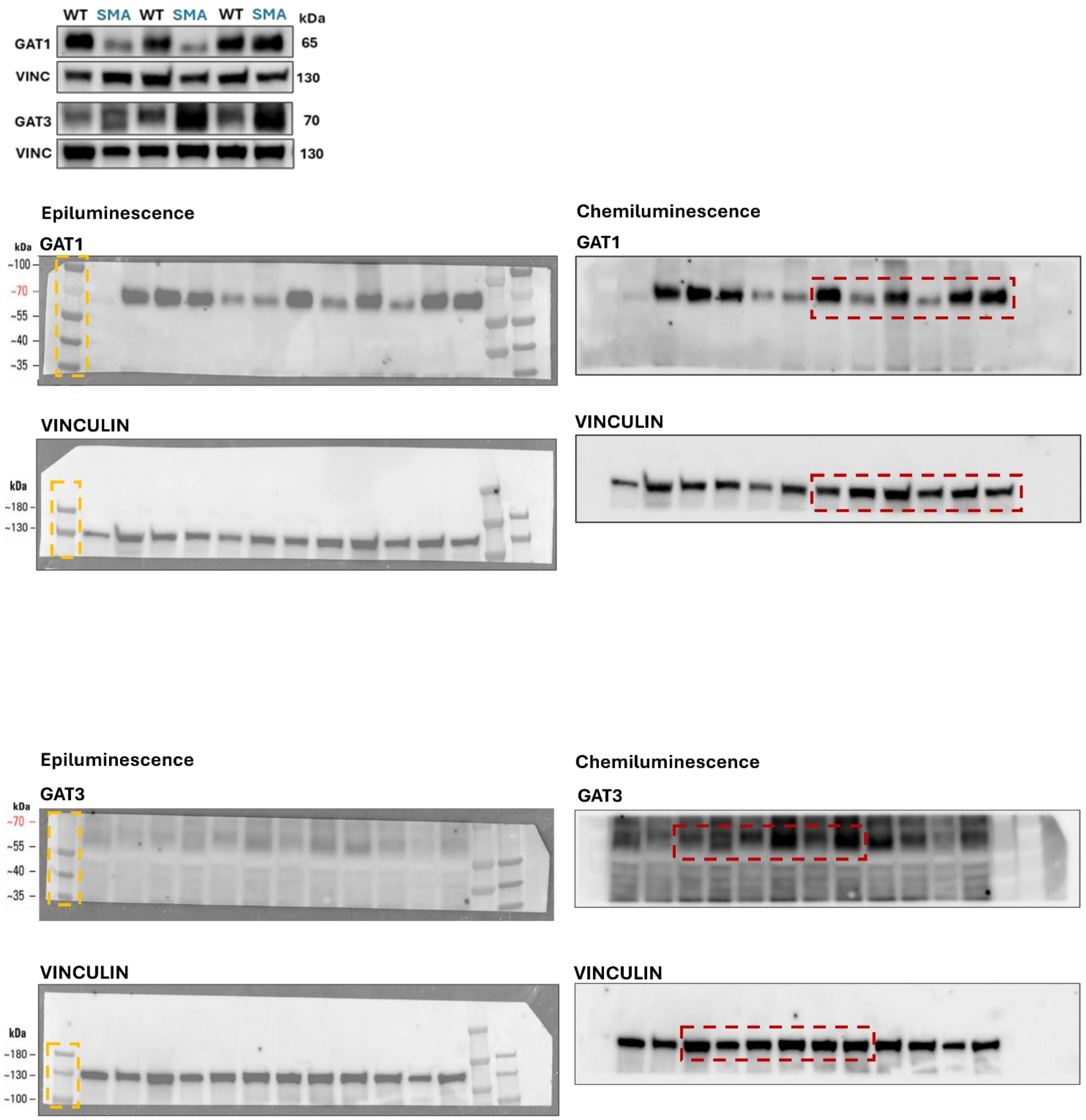
WT SMA WT SMA WT SMA kDa
GAT1 65
VINC 130
GAT3 70
VINC 130
Epiluminescence
kDa GAT1
~100
~70
~55
~40
~35
Chemiluminescence
GAT1
VINCULIN
kDa
~180
~130
VINCULIN
Epiluminescence
GAT3
kDa
~70
~55
~40
~35
Chemiluminescence
GAT3
VINCULIN
kDa
~180
~130
~100
VINCULIN

Figure 7A

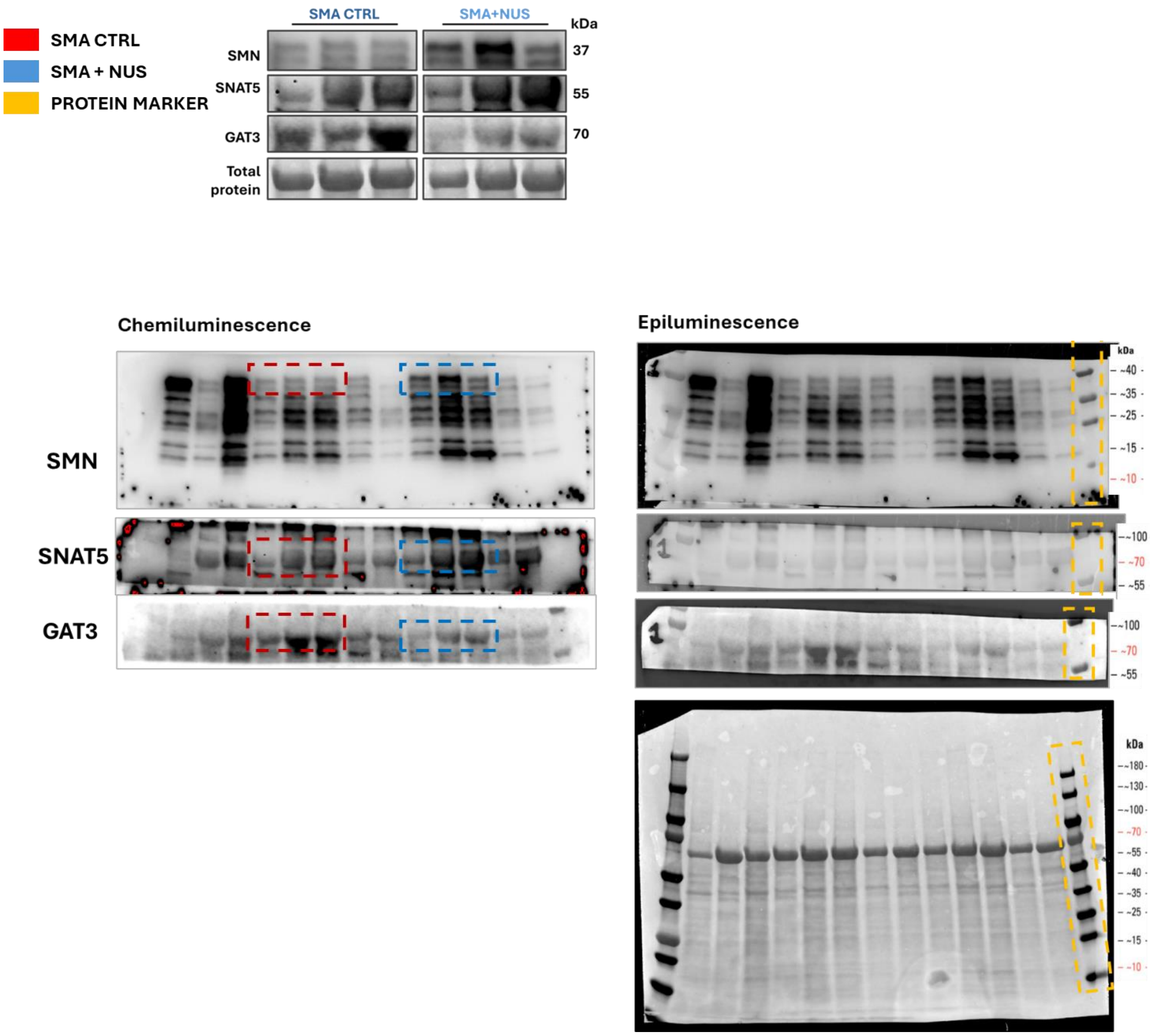

SMA CTRL
SMA + NUS
PROTEIN MARKER
SMA CTRL
SMA+NUS
kDa
SMN
SNAT5
GAT3
Total protein
37
55
70
Chemiluminescence
Epiluminescence
SMN
SNAT5
GAT3
kDa
~40
~35
~25
~15
~10
~100
~70
~55
~100
~70
~55
kDa
~180
~130
~100
~70
~55
~40
~35
~25
~15
~10

Supplementary Figure 2

a

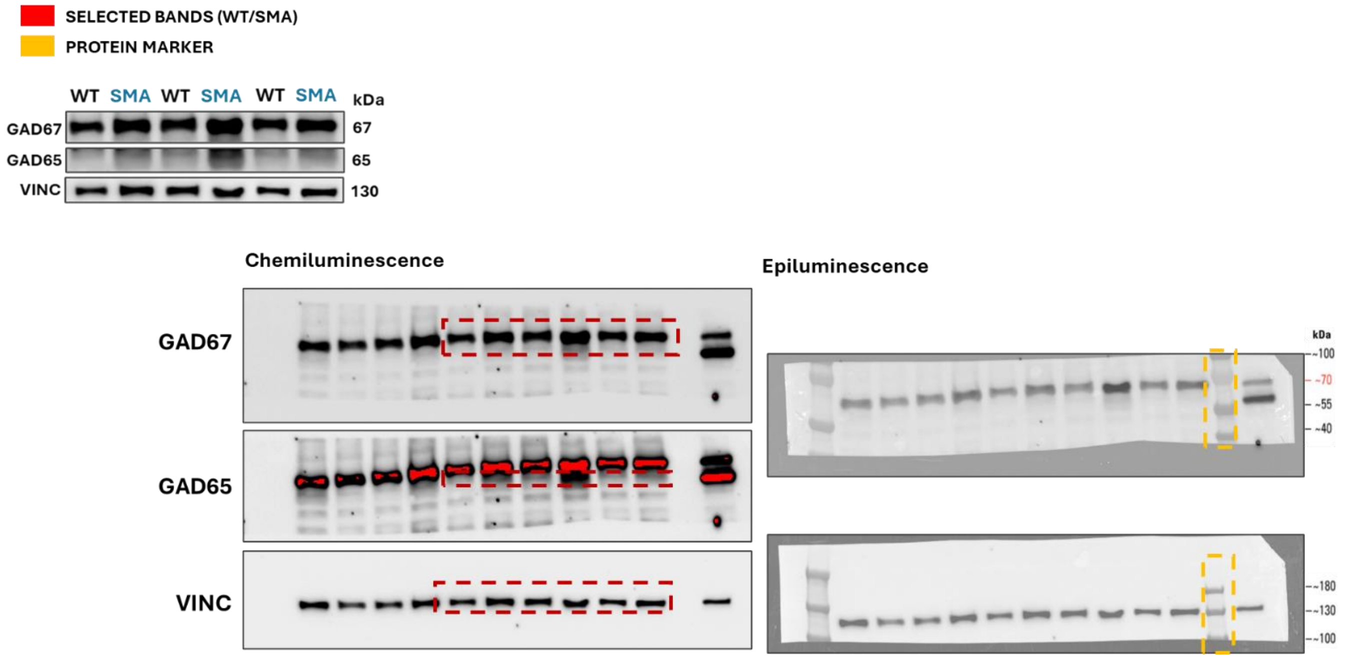

b

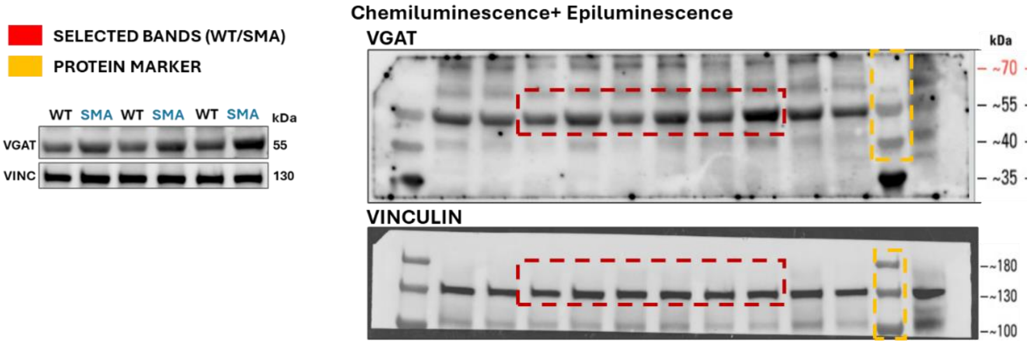

c

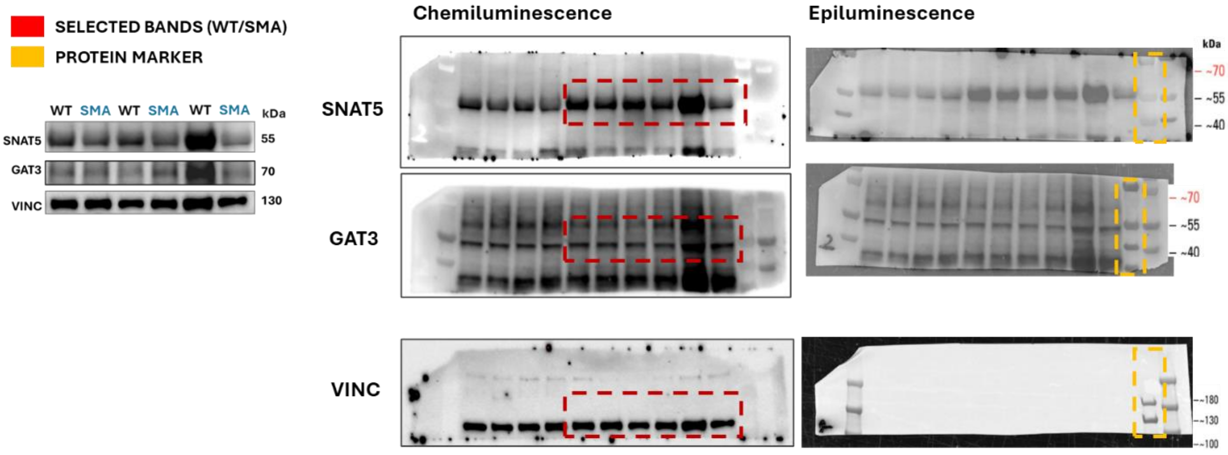

Supplementary Figure 4

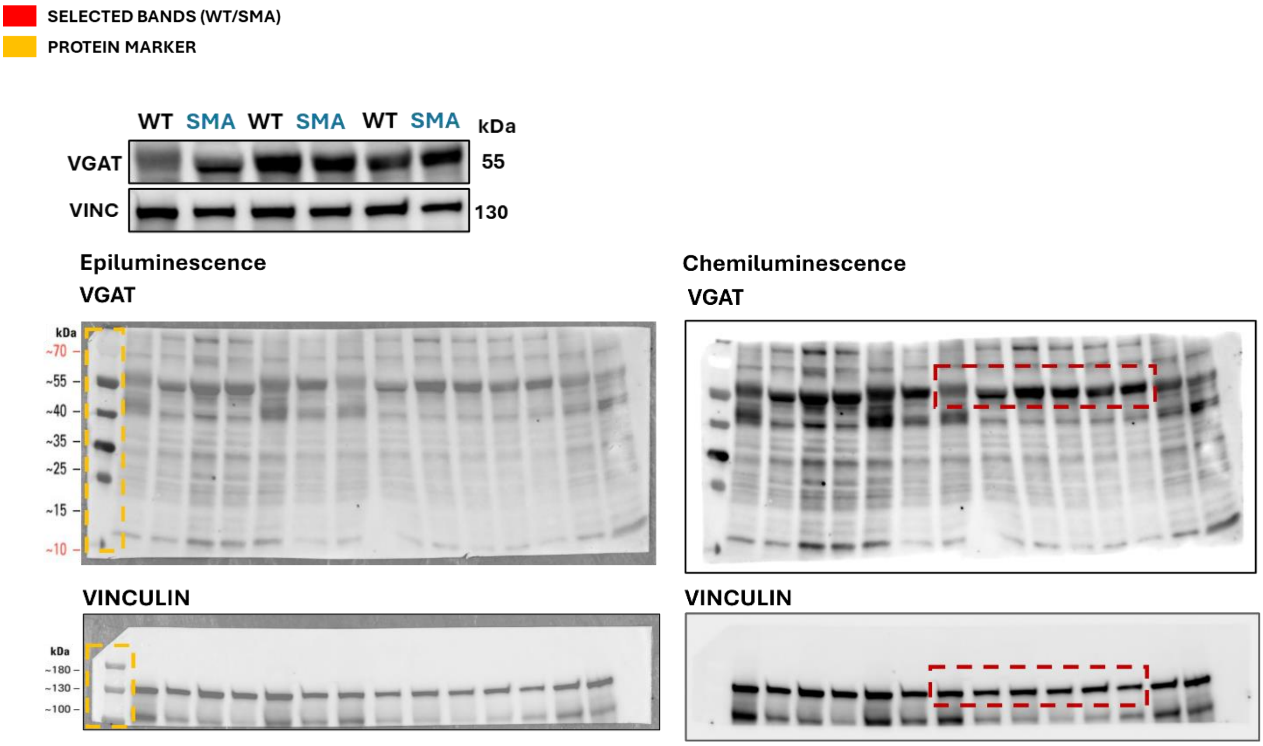

Supplement: Supplementary file 3 — Full unedited immunoblotting membranes [file 41419_2026_8520_MOESM3_ESM.pdf]
